# Checklist of *Sphagnum*-dwelling testate amoebae in Bulgaria

**DOI:** 10.3897/BDJ.6.e25295

**Published:** 2018-05-22

**Authors:** Nikola Bankov, Milcho Todorov, Anna Ganeva

**Affiliations:** 1 Institute of Biodiversity and Ecosystem Research, Bulgarian Academy of Sciences, Sofia, Bulgaria

**Keywords:** Amoebozoa, Cercozoa, Stramenopiles, biodiversity, distribution, *Sphagnum* mosses, Bulgaria

## Abstract

**Background:**

Until now, a complete checklist of *Sphagnum*-dwelling testate amoebae in Bulgaria has never been published. Records for species diversity and distribution in the country were scattered in many faunistic and ecological publications. The aim of the present study is to summarise all data for the species distribution at the level of country by reviewing the existing literature and by additional data obtained in our research over the past two years.

**New information:**

The checklist comprises 171 species, classified into 43 genera, 20 families, three orders, three classes and three phyla. We present data for 16 new *Sphagnum*-dwelling testate amoebae in Bulgaria and new distribution data for 134 species. Of them, 99 species are recorded from Stara Planina Mt., for which there was no available data to date. Additionally are recorded 69 new species for Pirin Mt., 21 for Vitosha Mt. and 18 for Rila Mt. Thirty six species are synonymised according to the latest taxonomic changes. Two misidentified taxa (*Euglypha brachiata* Penard, 1902 and Difflugia compressa var. africana Gauthier-Lièvre et Thomas, 1958) are transferred into valid species *E. acanthophora* and *Zivkovicia compressa*, respectively. Three of the recorded species have not been included in the checklist, because they are currently not refering to testate amoebae (*Cochliopodium bilimbosum* (Auerbach 1856) and *Cochliopodium echinatum* Korotneef, 1879 are gymnamoebae (naked amoebae) and *Microgromia elegantula* (Penard 1904) = *Paralieberkuehnia elegantula* (Penard 1904) is freshwater foraminifera).

## Introduction

Testate amoebae are a polyphyletic assemblage of at least three major, unrelated taxonomic groups of eukaryotes (Adl SM et al. 2012). They are present in most terrestrial and freshwater environments and more marginally in brackish and marine habitats. They are especially abundant and diverse in *Sphagnum* mosses. Research on testate amoebae have increased significantly over the past two decades due to their increasing use in different applied aspects: as a bioindicators for palaeoecological studies, in environmental monitoring, pollution hazards, ecotoxicology, studies on their role in the cycling of elements in terrestrial ecosystems, biogeographical and evolutionary studiesetc. (Aoki, Yoshiyuki et al. 2007, Booth 2002, Charman 2001, Charman et al. 1998, Lamentowicz and Mitchell 2005, Mitchell et al. 2008, Nguyen-Viet et al. 2007, Swindles et al. 2008, Swindles et al. 2015, Qin et al. 2013).

Studies on *Sphagnum*-dwelling testate amoebae in Bulgaria started at the beginning of the 20th century, when Pateff (1924), Pateff (1928) published his works on freshwater Rhizopoda in the country. He recorded a total of 118 rhizopods, of which 52 testate amoebae were from *Sphagnum* mosses in the Rila, Rhodopes and Vitosha Mountains. Later, Valkanov (1932), Valkanov (1934) published results of his studies on the fauna of alpine lakes in the Rila and Pirin Mountains and recorded many testate amoebae, which are typical inhabitants of peat mosses. Unfortunately, they were recorded along with all other species and no distinction was made amongst the species found in the lakes themselves and in the *Sphagnum* mosses from the lake shore. In the 60s and 70s of the 20th century, several publications on testate amoebae from the Vitosha, Rhodopes and Pirin Mountains were published (Golemansky 1965, Golemansky 1966, Golemansky 1968, Golemansky 1974), including a lot of data on *Sphagnum*-dwelling testate amoebae. At the end of the 20th and beginning of the 21th century, the number of publications concerning various aspects of taxonomy, systematics, morphology and ecology of sphagnicolous testate amoebae in Bulgaria increased considerably (Golemansky and Todorov 1990, Golemansky and Todorov 1993, Golemansky and Todorov 2006, Golemansky and Todorov 1985, Golemansky et al. 2006, Heger et al. 2010, Kosakyan et al. 2012, Todorov 1993, Todorov 2004, Todorov 2005, Todorov 2010, Todorov and Golemansky 1995, Todorov and Golemansky 2000, Todorov et al. 2010, Todorov et al. 2009). All data in the above-mentioned publications have never been summarised in one place and that is the reason why the main goal of the present paper is to unite this scattered information and, together with the new data obtained from our research over the past two years, to present a checklist of all known to date *Sphagnum*-dwelling testate amoebae in Bulgaria. Data on the distribution, habitats, regions, localities, altitude, authors and sources of information are provided in Suppl. material 1. Some typical and a few rare sphagnicolous testate amoebae, recorded from the country, are illustrated in Figs 1, 2, 3, 4.

## Materials and methods

The data presented in the checklist are based primarily on published information concerning *Sphagnum*-dwelling testate amoebae in Bulgaria. In addition, data from our research conducted over the past two years are also included. We have tried to explore the diversity and distribution of testate amoebae in the main areas of the country where peat mosses are more widespread. Genus *Sphagnum* in Bulgaria numbers 27 species (Natcheva and Ganeva 2005) distributed in mires in mountain areas of the country above the tree belt or in mires in coniferous forest, these being an important component of habitat 91D0 (Bog Woodland) of Directive 92/43/EEC. Some of the species (e.g. *Sphagnum capillifolium* (Ehrh.) Hedw., *S. subsecundum* Nees, *S. girgensohnii* Russ., *S. centrale* C.E.O.Jensen, *S. platyphyllum* (Lindb. ex. Braithw.) Sull. ex Warnst., *S. squarrosum* Crome, *S. teres* (Schimp.) Ängstr.) are more widespread in mires of Western Stara Planina, Rhodopes, Vitosha, Rila and Pirin Mountains, where, with other bryophytes and sedges, they comprise the specific mire flora.

The material for the present study was extracted from wet *Sphagnum* mosses, gathered at Vitosha and Western Stara Planina Mountains in 2016 and Rila and Pirin Mountains in 2017. A total of 109 samples from 18 localities were sampled and examined. All data, concerning sampling localities including date, region, altitude, coordinates, *Sphagnum* moss species, as well as many physical and chemical parameters of the sampling sites, are provided in Suppl. material 2.

Testate amoebae were extracted from fresh *Sphagnum* mosses at the sampling site and concentrated by sieving (350 μm). The resulting fraction (50 ml) was observed with an optical microscope “Amplival” (Zeiss-Jena) using 40x objective and 10x ocular lens. For scanning electron microscopy (SEM), specimens were isolated by searching through small isolates of material in a petri dish. Specimens were extracted using a glass micropipette, washed several times in distilled water and then individual shells were positioned with a single-hair brush on a previously mounted double-sided adhesive tape on a standard aluminium stub and air-dried. The shells were coated evenly with gold in a vacuum coating unit. The photomicrographs were obtained using a JEOL JSM-5510, operating at 10 kV.

The checklist is annotated with information regarding the synonymous names (if available), species distribution in *Sphagnum* mosses in Bulgaria, the relevant literature sources and notes (mainly taxonomic). The higher classification used here follows Adl SM et al. 2012 and Meisterfeld (2002a), Meisterfeld (2002b). Taxonomic revision of some genera and families, as well as numerous taxonomic and nomenclature changes, based on recent molecular studies and subsequent phylogenetic reconstructions, have also been taken into account (Blandenier et al. 2017, Chatelain et al. 2013, Gomaa et al. 2013, Gomaa et al. 2017, Heger et al. 2010, Kosakyan et al. 2016a, Kosakyan et al. 2012, Kosakyan et al. 2016b, Lahr et al. 2013, Lara et al. 2007, Mazei and Warren 2012, Mazei and Warren 2014, Mazei and Warren 2015).

The list does not include separate varieties and forms recorded from Bulgaria, despite the fact that, according to the International Code of Zoological Nomenclature, article 45.6.3, when the name was published before 1961 using the abbreviation ‘var.’ or ‘f.’, it is deemed to be subspecific rather than infrasubspecific. However, due to the fact that many of these taxa have not sufficiently detailed descriptions, which in many cases are based on signs that do not have much taxonomic significance (small differences in size, the presence or absence of spines/horns and their number, the number of lobes of the aperture etc.), these taxa remain with unclear taxonomic status. So, until the clarifying of their status with the help of combined morphological and molecular approaches and full confirmation of their validity, we prefere to adopt a conservative position and consider these taxa as the product of the phenotipic plasticity of nominal species. Nevertheless, in ‘Notes’ to each nominal species, we have included all the records for these infrasubspecific taxa, in the event that some of them may be raised in rank in the future.

## Checklists

### Checklist of *Sphagnum*-dwelling testate amoebae in Bulgaria

#### Protozoa

Owen,1858

#### Neozoa

Cavalier-Smith, 1993

#### Amoebozoa

Lühe, 1913, emend. Cavalier-Smith, 1998

#### Lobosa

Carpenter, 1861, emend. Cavalier-Smith, 2009

#### Tubulinea

Smirnov et al., 2005

#### Arcellinida

Kent, 1880

#### Arcellina

Haeckel, 1894

#### Microcoryciidae

de Saedeler, 1934

#### Microcorycia

Cockerell, 1911

#### Microcorycia flava

(Greeff, 1866), emend. Penard, 1902

Amphizonella flava Greeff, 1866
Corycia flava Greeff, 1866
Microcorycia flava *CoryciaDujardini*? Gagliardi, 1871 (in part)

##### Distribution

**Rhodopes Mt.** (Golemansky et al. 2006).

#### Microchlamyidae

Ogden, 1985

#### Microchlamys

Cockerell, 1911

#### Microchlamys patella

(Claparede and Lachmann, 1859) Cockerell, 1911

Pseudochlamys patella Claparede and Lachmann, 1859

##### Distribution

**Pirin Mt.** (new data); **Rhodopes Mt.** (Golemansky et al. 2006); **Rila Mt.** (Golemansky and Todorov 1993, Todorov and Golemansky 2000, Todorov 2004, Todorov 2005, new data); **Stara Planina Mt.** (new data); **Vitosha Mt.** (new data).

#### Arcellidae

Ehrenberg, 1843

#### Arcella

Ehrenberg, 1832

#### Arcella arenaria

Greeff, 1866

Arcella aureola Maggi, 1883
Arcella microstoma Penard, 1890

##### Distribution

**Pirin Mt.** (new data); **Rhodopes Mt.** (Golemansky et al. 2006); **Rila Mt.** (Golemansky and Todorov 1993, Todorov and Golemansky 2000, new data); **Vitosha Mt.** (Golemansky 1965, Golemansky and Todorov 1990, new data).

#### Arcella bathystoma

Deflandre, 1928

##### Distribution

**Rila Mt.** (new data).

#### Arcella catinus

Penard, 1890

Arcella vulgaris Leidy, 1879 (in part)
Arcella artocrea Penard, 1902
Arcella vulgaris var. compressa Cash, 1905
Arcella catinus var. australis Playfair, 1918

##### Distribution

**Pirin Mt.** (new data); **Rhodopes Mt.** (Golemansky 1968, Golemansky et al. 2006); **Rila Mt.** (Golemansky and Todorov 1993, new data); **Stara Planina Mt.** (new data); **Vitosha Mt.** (Pateff 1928, Golemansky 1965, Golemansky and Todorov 1990, Todorov 1993, Todorov and Golemansky 1995, new data).

#### Arcella dentata

Ehrenberg, 1830

Arcella stellaris Perty, 1852
Arcella Perty, 1852
Arcella stellata Ehrenberg, 1854

##### Distribution

**Pirin Mt.** (new data); **Rhodopes Mt.** (Pateff 1924, Golemansky et al. 2006).

#### Arcella discoides

Ehrenberg, 1871

Arcella discoidea Ehrenberg, 1843

##### Distribution

**Pirin Mt.** (Golemansky 1974, new data); **Rhodopes Mt.** (Pateff 1924, Golemansky 1968, Golemansky et al. 2006); **Rila Mt.** (Todorov and Golemansky 2000, Todorov 2004, Todorov 2005, new data); **Stara Planina Mt.** (new data); **Vitosha Mt.** (Pateff 1924, Golemansky 1965, Golemansky and Todorov 1985, Golemansky and Todorov 1990, Todorov 1993, Todorov and Golemansky 1995, new data).

##### Notes

Besides the nominal species, two infrasubspecific taxa have also been recorded: Arcella discoides var. pseudovulgaris Deflandre, 1928 (**Vitosha Mt.**) and Arcella discoides var. scutelliformis Playfair, 1918 (**Pirin Mt., Rhodopes Mt., Rila Mt.**).

#### Arcella gibbosa

Penard, 1890

##### Distribution

**Rhodopes Mt.** (Golemansky et al. 2006); **Vitosha Mt.** (Golemansky and Todorov 1985, Golemansky and Todorov 1990, Todorov 1993, Todorov and Golemansky 1995, new data).

#### Arcella hemisphaerica

Perty, 1852

Arcella vulgaris var. hemisphaerica Wailes, 1918

##### Distribution

**Pirin Mt.** (new data); **Rhodopes Mt.** (Golemansky 1968, Golemansky et al. 2006); **Rila Mt.** (Todorov 2004, new data); **Vitosha Mt.** (new data).

##### Notes

The species has been recorded both as nominal species and as infrasubspecific taxon *Arcella hemisphaerica* f. undulata Deflandre, 1928 (**Rhodopes Mt., Rila Mt.**).

#### Arcella intermedia

(Deflandre, 1928) Tsyganov and Mazei, 2006

Arcella hemisphaerica var. intermedia f. undulata Deflandre, 1928

##### Distribution

**Pirin Mt.** (new data); **Rila Mt.** (new data); **Stara Planina Mt.** (new data); **Vitosha Mt.** (new data).

#### Arcella rotundata

Playfair, 1918

##### Distribution

**Pirin Mt.** (Golemansky 1974, new data); **Rhodopes Mt.** (Golemansky et al. 2006); **Rila Mt.** (Todorov and Golemansky 2000, Todorov 2004, new data); **Stara Planina Mt.** (new data); **Vitosha Mt.** (Golemansky and Todorov 1985, Golemansky and Todorov 1990, Todorov 1993, Todorov and Golemansky 1995, new data).

##### Notes

Besides the nominal species, two infrasubspecific taxa have also been recorded: Arcella rotundata var. stenostoma Deflandre, 1928 (**Vitosha Mt.**) and Arcella rotundata var. stenostoma f. undulata Deflandre, 1928 (**Rhodopes Mt., Vitosha Mt.**).

#### Arcella vulgaris

Ehrenberg, 1830

##### Distribution

**Rhodopes Mt.** (Pateff 1924, Golemansky et al. 2006); **Rila Mt.** (Todorov and Golemansky 2000); **Vitosha Mt.** (Golemansky and Todorov 1985,Golemansky and Todorov 1990, Todorov 1993, Todorov and Golemansky 1995, new data).

##### Notes

The species has been recorded both as nominal species and as infrasubspecific taxon Arcella vulgaris f. undulata Deflandre, 1928 (**Rila Mt.**).

#### Netzeliidae

Kosakyan, Lara, Gomaa and Lahr, 2016

#### Netzelia

Ogden, 1979

#### Netzelia tuberculata

(Wallich, 1864) Netzel, 1983

Difflugia proteiformis subsp. globularis var. tuberculata Wallich, 1864
Difflugia tuberculata Archer, 1867
Difflugia lobostoma Leidy, 1879 (in part)
Nebela tuberculata Owen and Jones, 1976
Difflugia tricuspis Medioli and Scott, 1983 (in part)

##### Distribution

**Pirin Mt.** (new data); **Rhodopes Mt.** (Golemansky 1968, Golemansky et al. 2006); **Rila Mt.** (Todorov 2004).

##### Notes

The species has been recorded both as nominal species and as synonym *D. tuberculata* (**Rhodopes Mt.**).

#### Difflugina

Meisterfeld, 2002

#### Difflugiidae

Wallich, 1864

#### Difflugia

Leclerc, 1815

#### Difflugia acuminata

Ehrenberg, 1838

Difflugia curvicaulis Penard, 1899
Difflugia acuminata var. umbilicata Penard, 1902
Difflugia venusta Ogden, 1983

##### Distribution

**Pirin Mt.** (new data); **Rhodopes Mt.** (Pateff 1924, Golemansky 1968, Golemansky et al. 2006); **Rila Mt.** (Todorov and Golemansky 2000, Todorov 2004, Todorov 2005, new data); **Vitosha Mt.** (Pateff 1924, new data).

##### Notes

The species has been recorded both as nominal species and as synonym *D. venusta* (**Rhodopes Mt.**).

#### Difflugia ampullula

Playfair, 1918

##### Distribution

**Pirin Mt.** (new data); **Rila Mt.** (Todorov 2005, new data); **Vitosha Mt.** (new data).

#### Difflugia angulostoma

Gauthier-Lièvre and Thomas, 1958

##### Distribution

**Rila Mt.** (Todorov and Golemansky 2000); **Stara Planina Mt.** (new data).

#### Difflugia bacillariarum

Perty, 1849

Difflugia bicornis Penard, 1890
Difflugia australis var. minor Gauthier-Lièvre et Thomas, 1958

##### Distribution

**Stara Planina Mt.** (new data); **Rila Mt.** (Golemansky and Todorov 1993).

#### Difflugia brevicolla

Cash, 1909

Difflugia pyriformis var. atricolor Penard, 1902

##### Distribution

**Rhodopes Mt.** (Golemansky et al. 2006); **Rila Mt.** (new data); **Stara Planina Mt.** (new data).

#### Difflugia bryophila

(Penard, 1902) Jung, 1942

Difflugia pyriformis var. bryophila Penard, 1902
Difflugia oblonga var. longicollis Gassowsky, 1936
Difflugia longicollis (Gassowsky, 1936) Ogden and Hedley, 1980
Difflugia gassowskii Ogden, 1983

##### Distribution

**Pirin Mt.** (new data); **Rhodopes Mt.** (Golemansky et al. 2006); **Rila Mt.** (Todorov and Golemansky 2000, Todorov 2004, Todorov 2005, new data); **Stara Planina Mt.** (new data); **Vitosha Mt.** (Golemansky and Todorov 1985, Golemansky and Todorov 1990, Todorov 1993, Todorov and Golemansky 1995, new data).

##### Notes

The species has been recorded both as nominal species and as synonym *D. gassowskii* (**Rhodopes Mt., Rila Mt., Vitosha Mt.**).

#### Difflugia distenda

Ogden, 1983

Difflugia acuminata var. inflata Penard, 1899
Difflugia bicruris Gauthier-Lièvre et Thomas, 1958.

##### Distribution

**Pirin Mt.** (new data); **Vitosha Mt.** (Todorov 1993, Todorov and Golemansky 1995).

#### Difflugia elegans

Penard, 1890

Difflugia amphoralis Hopkinson, 1909
Difflugia australis (Playfair, 1918) Gauthier-Lièvre et Thomas, 1958
Difflugia borodini Gassowsky, 1936
Difflugia elegans f. bicornis Jung, 1936
Difflugia elegans f. tricornis Jung, 1936
Difflugia juzephiniensis Dekhtyar, 1993
Difflugia leidyi Wailes, 1912
Difflugia Mereschkowsky, 1877
Difflugia tricornis (Jung, 1936) Ogden, 1983
Difflugia varians Penard, 1902

##### Distribution

**Pirin Mt.** (Golemansky 1974, new data); **Rhodopes Mt.** (Golemansky 1968, Golemansky et al. 2006); **Rila Mt.** (Pateff 1924, Todorov and Golemansky 2000, Todorov 2004, Todorov 2005, new data); **Stara Planina Mt.** (new data); **Vitosha Mt.** (Pateff 1924, Golemansky 1965, Todorov 1993, Todorov and Golemansky 1995, new data).

##### Notes

The species has been recorded both as nominal species and as synonym *D. amphoralis* (**Rila Mt., Vitosha Mt.**).

#### Difflugia glans

Penard, 1902

##### Distribution

**Rila Mt.** (Todorov and Golemansky 2000).

#### Difflugia globulosa

Dujardin, 1837

Difflugia proteiformis Ehrenberg, 1838 subsp. globularis Wallich, 1864
Difflugia globularis (Wallich, 1864) Leidy, 1877
Difflugia chardezi Godeanu, 1972

##### Distribution

**Pirin Mt.** (new data); **Rhodopes Mt.** (Golemansky et al. 2006); **Rila Mt.** (Todorov and Golemansky 2000, Todorov 2004, Todorov 2005, new data); **Stara Planina Mt.** (new data); **Vitosha Mt.** (Todorov 1993, Golemansky and Todorov 1985, Golemansky and Todorov 1990, Todorov and Golemansky 1995, new data).

##### Notes

The species has been recorded both as nominal species and as synonym *D. globularis* (**Rhodopes Mt., Rila Mt., Vitosha Mt.**).

#### Difflugia hiraethogii

Ogden, 1983

##### Distribution

**Pirin Mt.** (new data); **Rila Mt.** (Todorov and Golemansky 2000, Todorov 2004, Todorov 2005, new data); **Stara Planina Mt.** (new data); **Vitosha Mt.** (new data).

#### Difflugia lanceolata

Penard,1890

##### Distribution

**Pirin Mt.** (new data); **Rhodopes Mt.** (Golemansky 1968, Golemansky et al. 2006); **Rila Mt.** (Todorov and Golemansky 2000, new data); **Stara Planina Mt.** (new data); **Vitosha Mt.** (Golemansky 1965, Golemansky and Todorov 1990).

#### Difflugia lobostoma

Leidy, 1874

##### Distribution

**Rhodopes Mt.** (Golemansky 1968, Golemansky et al. 2006)

#### Difflugia lucida

Penard, 1890

##### Distribution

**Pirin Mt.** (Golemansky 1974, new data); **Rhodopes Mt.** (Golemansky et al. 2006); **Rila Mt.** (Golemansky and Todorov 1993, Todorov and Golemansky 2000, Todorov 2004, Todorov 2005, new data); **Stara Planina Mt.** (new data); **Vitosha Mt.** (Golemansky 1965, Golemansky and Todorov 1985, Golemansky and Todorov 1990, Todorov 1993, Todorov and Golemansky 1995, new data).

#### Difflugia mammillaris

Penard, 1893

##### Distribution

**Rila Mt.** (Todorov 2004).

#### Difflugia mica

Frenzel, 1892

##### Distribution

**Rila Mt.** (Todorov 2005); **Vitosha Mt.** (new data).

#### Difflugia microclaviformis

(Kourov, 1925) Ogden, 1983

Difflugia pyriformis var. venusta Penard 1902
Difflugia oblogna var. venusta Cash, 1909
Difflugia oblonga var. microclaviformis Kourov, 1925

##### Distribution

**Vitosha Mt.** (Golemansky 1965, Golemansky and Todorov 1990).

##### Notes

The species has been recorded both as nominal species and as the infrasubspecific taxon Difflugia oblonga var. microclaviformis (**Vitosha Mt.**).

#### Difflugia minuta

Rampi, 1950

##### Distribution

**Pirin Mt.** (new data); **Rila Mt.** (Todorov 2004, Todorov 2005); **Vitosha Mt.** (new data).

#### Difflugia molesta

Penard, 1902

Difflugia levanderi Playfair, 1918

##### Distribution

**Rhodopes Mt.** (Golemansky et al. 2006); **Rila Mt.** (Todorov 2004, Todorov 2005, new data); **Vitosha Mt.** (new data).

#### Difflugia oblonga

Ehrenberg, 1838

Difflugia bacillifera Penard, 1890
Difflugia lacustris (Penard, 1899) Ogden, 1983
Difflugia oblonga f. cyphodera Jung, 1942
Difflugia oblonga var. incondita Gauthier-Lièvre et Thomas, 1958
Difflugia oblonga var. lacustris Cash and Hopkinson, 1909
Difflugia oblonga var. parva Thomas, 1954
Difflugia parva (Thomas, 1954) Ogden, 1983
Difflugia pyriformis var. lacustris Penard, 1899

##### Distribution

**Pirin Mt.** (new data); **Rhodopes Mt.** (Golemansky 1968, Golemansky et al. 2006); **Rila Mt.** (Pateff 1924, Todorov and Golemansky 2000, Todorov 2005, new data); **Stara Planina Mt.** (new data); **Vitosha Mt.** (Pateff 1924, Golemansky 1965, Golemansky and Todorov 1985, Golemansky and Todorov 1990, Todorov 1993, Todorov and Golemansky 1995, new data).

##### Notes

Besides the nominal species, the synonyms *D. parva* (**Rhodopes Mt., Rila Mt., Vitosha Mt.**), *D. lacustris* (**Rhodopes Mt., Vitosha Mt.**), *D. bacillifera* (**Rila Mt., Vitosha Mt.**) and infrasubspecific taxa D. oblonga var. lacustris (Vitosha Mt.) and D. oblonga var. parva (**Rila Mt., Vitosha Mt.**) have also been recorded.

#### Difflugia penardi

Hopkinson,1909

Difflugia fallax Penard, 1890
Difflugia pyriformis var. tenuis Penard, 1890
Difflugia manicata Penard, 1902
Difflugia oblonga var. tenuis Wailes and Penard, 1911
Difflugia tenuis (Penard, 1890) Ogden, 1983

##### Distribution

**Pirin Mt.** (Golemansky 1974, new data); **Rhodopes Mt.** (Golemansky 1968, Golemansky et al. 2006); **Rila Mt.** (Todorov and Golemansky 2000, Todorov 2004, new data); **Stara Planina Mt.** (new data); **Vitosha Mt.** (Golemansky 1965, Golemansky and Todorov 1990, Todorov 1993, Todorov and Golemansky 1995, new data).

##### Notes

The species has been recorded both as nominal species and as synonym *D. manicata* (**Rhodopes Mt., Rila Mt., Vitosha Mt.**).

#### Difflugia petricola

Cash, 1909

##### Distribution

**Pirin Mt.** (new data); **Rhodopes Mt.** (Golemansky et al. 2006); **Rila Mt.** (Todorov 2004, Todorov 2005).

#### Difflugia pristis

Penard, 1902

##### Distribution

**Pirin Mt.** (new data); **Rhodopes Mt.** (Golemansky et al. 2006); **Rila Mt.** (Todorov and Golemansky 2000, Todorov 2004, Todorov 2005, new data); **Stara Planina Mt.** (new data); **Vitosha Mt.** (new data).

#### Difflugia pulex

Penard, 1902

Difflugia minuta var. minor Godeanu, 1972
Difflugia ovalisina Beyens et Chardez, 1994

##### Distribution

**Pirin Mt.** (new data); **Rhodopes Mt.** (Golemansky et al. 2006); **Rila Mt.** (Pateff 1924, Todorov and Golemansky 2000, Todorov 2004, Todorov 2005, new data); **Stara Planina Mt.** (new data); **Vitosha Mt.** (Golemansky and Todorov 1990, Todorov 1993, Todorov and Golemansky 1995, new data).

#### Difflugia pyriformis

Perty, 1849

##### Distribution

**Rhodopes Mt.** (Pateff 1924); **Rila Mt.** (Pateff 1924); **Vitosha Mt.** (Pateff 1924).

#### Difflugia rotunda

(Chardez, 1956) Ogden, 1983

Difflugia globularis var. sphaerica Chardez, 1956

##### Distribution

**Rila Mt.** (Todorov 2004).

#### Difflugia rubescens

Penard, 1891

##### Distribution

**Pirin Mt.** (Golemansky 1974, new data); **Rila Mt.** (Pateff 1924, Golemansky and Todorov 1993, new data); **Stara Planina Mt.** (new data); **Vitosha Mt.** (Golemansky 1965, Golemansky and Todorov 1985, Golemansky and Todorov 1990, Todorov 1993, Todorov and Golemansky 1995, new data).

#### Difflugia stoutii

Ogden, 1983

##### Distribution

**Rila Mt.** (Todorov and Golemansky 2000).

#### Difflugia subaequalis

Penard, 1910

##### Distribution

**Pirin Mt.** (Golemansky 1974).

#### Difflugia urceolata

Carter, 1864

Difflugia urceolata var. olla Leidy, 1879
Difflugia urceolata var. sphaerica Playfair, 1917

##### Distribution

**Pirin Mt.** (new data); **Rhodopes Mt.** (Pateff 1924, Golemansky et al. 2006).

#### Difflugia ventricosa

Deflandre, 1926

##### Distribution

**Rhodopes Mt.** (Golemansky et al. 2006).

#### Difflugia viscidula

Penard, 1902

Difflugia lemani Blanc, 1892
Difflugia histrio Penard, 1908
Difflugia lemani var. palustris Chardez, 1956
Difflugia lebes var. masurica Schönborn, 1965
Difflugia lebes var. bretschkoi Laminger, 1971
Difflugia finstertaliensis Laminger, 1971

##### Distribution

**Pirin Mt.** (new data); **Rhodopes Mt.** (Golemansky et al. 2006); **Rila Mt.** (Todorov and Golemansky 2000, Todorov 2004, Todorov 2005, new data); **Vitosha Mt.** (Pateff 1928, Golemansky 1965, Golemansky and Todorov 1985, Golemansky and Todorov 1990).

##### Notes

The species has been recorded as nominal species, as synonym *D. lemani* (**Vitosha Mt.**), as infrasubspecific taxon D. lemani var. palustris (**Vitosha Mt.**) and as *Difflugia* sp. (**Vitosha Mt.**).

#### Lagenodifflugia

Medioli and Scott, 1983

#### Lagenodifflugia bryophila

(Penard, 1902) Ogden, 1987

Pontigulasia bryophila Penard, 1902
Pontigulasia bryophila var. elachys Jung, 1942
Pontigulasia varadi Godeanu, 1972
Zivkovicia bryophila (Penard, 1902) Ogden, 1983

##### Distribution

**Pirin Mt.** (Golemansky 1974, new data); **Rhodopes Mt.** (Pateff 1924, Golemansky et al. 2006); **Rila Mt.** (Pateff 1924, Todorov and Golemansky 2000, Todorov 2004, Todorov 2005, new data); **Stara Planina Mt.** (new data); **Vitosha Mt.** (Pateff 1924, Golemansky 1965, Golemansky and Todorov 1990, Todorov 1993, Todorov and Golemansky 1995, new data).

##### Notes

The species has been recorded both as nominal species and as synonym *P. bryophila* (**Rhodopes Mt., Rila Mt., Vitosha Mt.**).

#### Lagenodifflugia montana

(Ogden and Zivkovic, 1983) Ogden, 1987

Pontigulasia montana Ogden and Zivkovic, 1983

##### Distribution

**Pirin Mt.** (new data); **Rila Mt.** (new data).

#### Lagenodifflugia vas

(Leidy, 1874) Medioli and Scott, 1983

Difflugia vas Leidy, 1874
Difflugia pyriformis var. vas Leidy, 1879
Pontigulasia vas (Leidy) Schouteden, 1906
Zivkovicia vas (Leidy, 1874) Ogden, 1983 (in part)

##### Distribution

**Pirin Mt.** (new data); **Rhodopes Mt.** (Golemansky et al. 2006); **Rila Mt.** (new data); **Vitosha Mt.** (new data).

#### Pontigulasia

Rhumbler, 1896

#### Pontigulasia elisa

(Penard, 1893) Schouteden, 1906

Difflugia elisa Penard, 1893
Pontigulasia incisa Rhumbler, 1896

##### Distribution

**Pirin Mt.** (new data); **Rila Mt.** (Golemansky and Todorov 1993); **Vitosha Mt.** (Todorov 1993, Todorov and Golemansky 1995).

#### Pontigulasia rhumbleri

Hopkinson, 1920

Pontigulasia compressa Rhumbler, 1896
Pontigulasia rhumbleri (non *Pontigulasia compressa* (Carter, 1864))

##### Distribution

**Pirin Mt.** (new data); **Rhodopes Mt.** (Pateff 1924, Golemansky et al. 2006); **Rila Mt.** (Pateff 1924, Todorov 2004, Todorov 2005); **Stara Planina Mt.** (new data); **Vitosha Mt.** (Pateff 1924).

##### Notes

The species has been recorded both as nominal species and as synonym *P. compressa* (**Rhodopes Mt., Rila Mt., Vitosha Mt.**).

#### Zivkovicia

Ogden, 1987

#### Zivkovicia compressa

(Carter, 1864)

Difflugia compressa Carter, 1864
Difflugia pyriformis var. vas sub- var. bigibbosa Penard, 1899
Pontigulasia bigibbosa Penard, 1902

##### Distribution

**Pirin Mt.** (Golemansky 1974, new data); **Rhodopes Mt.** (Golemansky et al. 2006); **Rila Mt.** (Todorov and Golemansky 2000, Todorov 2004, Todorov 2005, new data); **Stara Planina Mt.** (new data); **Vitosha Mt.** (Golemansky 1965, Golemansky and Todorov 1985, Golemansky and Todorov 1990, Todorov 1993, Todorov and Golemansky 1995, new data).

##### Notes

The species has been recorded both as nominal species and as synonym *P. bigibbosa* (**Pirin Mt., Vitosha Mt.**). Furthermore, in several publications on the testate amoebae from **Vitosha Mt.** (Golemansky 1965, Golemansky and Todorov 1990, Todorov 1993, Todorov and Golemansky 1995), the infrasubspecific taxa Difflugia compressa var. africana Gauthier-Lièvre et Thomas, 1958 has erroneously been recorded, because the description of the found individuals fully corresponds to *Z. compressa*.

#### Zivkovicia spectabilis

(Penard, 1902) Ogden, 1987

Pontigulasia spectabilis Penard, 1902
Zivkovicia vas (Leidy, 1874) Ogden, 1983 (in part)

##### Distribution

**Rhodopes Mt.** (Golemansky 1968); **Rila Mt.** (Todorov 2004).

##### Notes

The species has been recorded both as nominal species and as synonym *P. spectabilis* (**Rhodopes Mt.**).

#### Centropyxidae

Jung, 1942

#### Centropyxis

Stein, 1857

#### Centropyxis aculeata

(Ehrenberg, 1830) Stein, 1857

Arcella aculeata Ehrenberg, 1830
Difflugia aculeata Perty, 1852
Echinopyxis aculeata Claparède et Lachmann, 1859

##### Distribution

**Pirin Mt.** (Golemansky 1974, new data); **Rhodopes Mt.** (Pateff 1924, Golemansky 1968, Golemansky et al. 2006); **Rila Mt.** (Golemansky and Todorov 1993, Todorov and Golemansky 2000, Todorov 2004, Todorov 2005, new data); **Stara Planina Mt.** (new data); **Vitosha Mt.** (Pateff 1924, Golemansky 1965, Golemansky and Todorov 1985, Golemansky and Todorov 1990, Todorov 1993, Todorov and Golemansky 1995, new data).

##### Notes

Besides the nominal species, two infrasubspecific taxa Centropyxis aculeata var. oblonga Deflandre, 1929 (**Rhodopes Mt., Rila Mt., Vitosha Mt.**) and Centropyxis aculeata var. grandis Deflandre, 1929 **(Vitosha Mt.**) have also been recorded.

#### Centropyxis aerophila

Deflandre, 1929

Difflugia constricta Ehrenberg, 1838
Arcella arctiscon Ehrenberg, 1854

##### Distribution

**Pirin Mt.** (Golemansky 1974, new data); **Rhodopes Mt.** (Golemansky 1968, Golemansky et al. 2006); **Rila Mt.** (Golemansky and Todorov 1993, Todorov and Golemansky 2000, Todorov 2004, Todorov 2005, new data); **Stara Planina Mt.** (new data); **Vitosha Mt.** (Golemansky 1965, Golemansky and Todorov 1985, Golemansky and Todorov 1990, Todorov 1993, Todorov and Golemansky 1995, new data).

##### Notes

Besides the nominal species, the infrasubspecific taxon Centropyxis aerophila var. sphagnicola Deflandre, 1929 has also been recorded (**Rila Mt., Vitosha Mt.**).

#### Centropyxis cassis

(Wallich, 1864) Deflandre, 1929

Difflugia cassis Wallich, 1864

##### Distribution

**Pirin Mt.** (new data); **Rhodopes Mt.** (Golemansky et al. 2006); **Rila Mt.** (Todorov 2004, Todorov 2005, Todorov and Golemansky 2000, new data); **Stara Planina Mt.** (new data); **Vitosha Mt.** (Golemansky 1965, Golemansky and Todorov 1985, Golemansky and Todorov 1990, Todorov 1993, Todorov and Golemansky 1995, new data).

#### Centropyxis constricta

(Ehrenberg, 1838) Penard, 1902

Difflugia constricta Ehrenberg, 1838
Arcella constricta Ehrenberg, 1841

##### Distribution

**Pirin Mt.** (Golemansky 1974, new data); **Rhodopes Mt.** (Pateff 1924, Golemansky et al. 2006); **Rila Mt.** (Pateff 1924, new data); **Stara Planina Mt.** (new data); **Vitosha Mt.** (Pateff 1924, Golemansky 1965, Golemansky and Todorov 1990, new data).

##### Notes

The species has been recorded both as nominal species and as synonym *D. constricta* (**Rhodopes Mt., Rila Mt., Vitosha Mt.**).

#### Centropyxis cryptostoma

Bonnet, 1959

##### Distribution

**Rila Mt.** (new data).

#### Centropyxis discoides

(Penard, 1890) Deflandre, 1929

Centropyxis aculeata var. discoides Penard, 1890

##### Distribution

**Pirin Mt.** (new data); **Rhodopes Mt.** (Golemansky et al. 2006); **Rila Mt.** (Todorov 2005, new data); **Stara Planina Mt.** (new data); **Vitosha Mt.** (Golemansky 1965, Golemansky and Todorov 1990, new data).

#### Centropyxis ecornis

(Ehrenberg, 1841) Leidy, 1879

Arcella ecornis Ehrenberg, 1841

##### Distribution

**Pirin Mt.** (Golemansky 1974, new data); **Rhodopes Mt.** (Golemansky et al. 2006); **Rila Mt.** (Todorov and Golemansky 2000, Todorov 2004, Todorov 2005, new data); **Stara Planina Mt.** (new data); **Vitosha Mt.** (Todorov 1993, Todorov and Golemansky 1995, new data).

#### Centropyxis elongata

(Penard, 1890) Thomas, 1959

Difflugia constricta var. elongata Penard, 1890

##### Distribution

**Pirin Mt.** (Golemansky 1974, new data); **Rhodopes Mt.** (Golemansky et al. 2006); **Rila Mt.** (Todorov and Golemansky 2000, new data); **Vitosha Mt.** (Golemansky and Todorov 1985, Golemansky and Todorov 1990, Todorov 1993, Todorov and Golemansky 1995).

#### Centropyxis gibba

Deflandre, 1929

##### Distribution

**Rhodopes Mt.** (Golemansky et al. 2006); **Rila Mt.** (Todorov 2005).

#### Centropyxis hirsuta

Deflandre, 1929

##### Distribution

**Rila Mt.** (Todorov 2004).

#### Centropyxis laevigata

Penard, 1890

##### Distribution

**Rhodopes Mt.** (Golemansky et al. 2006).

#### Centropyxis minuta

Deflandre, 1929

Difflugia constricta p. p. Leidy, 1879, PL. XVIII, figs. 15-16
Difflugia constricta p. p. Penard, 1902, p. 299, figs. 13-14

##### Distribution

**Rhodopes Mt.** (Golemansky et al. 2006).

#### Centropyxis orbicularis

Deflandre, 1929

##### Distribution

**Pirin Mt.** (new data); **Rila Mt.** (new data); **Stara Planina Mt.** (new data).

#### Centropyxis plagiostoma

Bonnet and Thomas, 1955

##### Distribution

**Rhodopes Mt.** (Golemansky et al. 2006); **Rila Mt.** (Golemansky and Todorov 1993).

#### Centropyxis platystoma

(Penard, 1890) Deflandre, 1929

Difflugia platystoma Penard, 1890
Difflugia constricta p. p. Leidy, 1879, PL. XVIII, figs. 20-21
Difflugia constricta p. p. Penard, 1902, p. 299, figs. 8, 11, 12

##### Distribution

**Pirin Mt.** (new data); **Rhodopes Mt.** (Golemansky et al. 2006); **Rila Mt.** (Todorov and Golemansky 2000, Todorov 2005, new data); **Stara Planina Mt.** (new data); **Vitosha Mt.** (Golemansky 1965, Golemansky and Todorov 1985, Golemansky and Todorov 1990, Todorov 1993, Todorov and Golemansky 1995, new data).

##### Notes

Besides the nominal species, the infrasubspecific taxon Centropyxis platystoma var. armata Deflandre, 1929 has also been recorded (**Vitosha Mt.**).

#### Centropyxis spinosa

(Cash, 1905) Deflandre, 1929

Centropyxis aculeata var. spinosa Cash, 1905

##### Distribution

**Pirin Mt.** (new data); **Vitosha Mt.** (Todorov 1993, Todorov and Golemansky 1995).

#### Centropyxis sylvatica

(Deflandre, 1929) Bonnet and Thomas, 1955

Centropyxis aerophila var. sylvatica Deflandre, 1929

##### Distribution

**Pirin Mt.** (Golemansky 1974, new data); **Rhodopes Mt.** (Golemansky et al. 2006); **Rila Mt.** (Golemansky and Todorov 1993, Todorov 2004, Todorov 2005, new data); **Stara Planina Mt.** (new data); **Vitosha Mt.** (Golemansky 1965, Golemansky and Todorov 1985, Golemansky and Todorov 1990, Todorov 1993, Todorov and Golemansky 1995, new data).

#### Trigonopyxidae

Loeblich and Tappan, 1964

#### Cyclopyxis

Deflandre, 1929

#### Cyclopyxis arcelloides

(Penard, 1902) Deflandre, 1929

Centropyxis arceloides Penard, 1902

##### Distribution

**Vitosha Mt.** (Pateff 1928, Golemansky 1965, Golemansky and Todorov 1990, new data).

##### Notes

The species is recorded both as nominal species and as synonym *Centropyxis arceloides* (**Vitosha Mt.)**.

#### Cyclopyxis eurystoma

Deflandre, 1929

Centropyxis (Cyclopyxis) eurystoma Deflandre, 1929

##### Distribution

**Pirin Mt.** (Golemansky 1974, new data); **Rhodopes Mt.** (Golemansky 1968, Golemansky et al. 2006); **Rila Mt.** (Golemansky and Todorov 1993, Todorov and Golemansky 2000, new data); **Stara Planina Mt.** (new data); **Vitosha Mt.** (Golemansky 1965, Golemansky and Todorov 1985, Golemansky and Todorov 1990, Todorov 1993, Todorov and Golemansky 1995, new data).

#### Cyclopyxis kahli

Deflandre, 1929

Centropyxis (Cyclopyxis) kahli Deflandre, 1929

##### Distribution

**Pirin Mt.** (Golemansky 1974, new data); **Rhodopes Mt.** (Golemansky et al. 2006); **Rila Mt.** (Golemansky and Todorov 1993, Todorov and Golemansky 2000, new data); **Stara Planina Mt.** (new data); **Vitosha Mt.** (Golemansky 1965, Golemansky and Todorov 1985, Golemansky and Todorov 1990, new data).

#### Cyclopyxis pirini

Golemansky, 1974

##### Distribution

**Pirin Mt.** (Golemansky 1974); **Vitosha Mt.** (Golemansky and Todorov 1985, Golemansky and Todorov 1990, Todorov 1993, Todorov and Golemansky 1995, new data).

#### Cyclopyxis puteus

Thomas, 1960

##### Distribution

**Rila Mt.** (new data).

#### Trigonopyxis

Penard, 1912

#### Trigonopyxis arcula

(Leidy, 1879) Penard, 1912

Difflugia arcula Leidy, 1879
Cystidina arcula (Leidy, 1879) Volz, 1929

##### Distribution

**Rila Mt.** (new data); **Stara Planina Mt.** (new data); **Vitosha Mt.** (Golemansky 1965, Golemansky and Todorov 1990, new data).

#### Plagiopyxidae

Bonnet and Thomas, 1960

#### Bullinularia

Deflandre, 1953

#### Bullinularia indica

(Penard, 1907) Deflandre, 1953

Bulinella indica Penard, 1907
Bullinula indica Penard, 1912

##### Distribution

**Pirin Mt.** (new data); **Rila Mt.** (new data).

#### Plagiopyxis

Penard, 1910

#### Plagiopyxis callida

Penard, 1910

Bullinula indica var. callida Jung, 1936

##### Distribution

**Pirin Mt.** (Golemansky 1974, new data); **Rila Mt.** (new data); **Stara Planina Mt.** (new data); **Vitosha Mt.** (new data).

#### Plagiopyxis declivis

Thomas, 1955

##### Distribution

**Pirin Mt.** (new data); **Rhodopes Mt.** (Golemansky et al. 2006); **Rila Mt.** (Todorov and Golemansky 2000, new data); **Stara Planina Mt.** (new data); **Vitosha Mt.** (Todorov 1993, Todorov and Golemansky 1995, new data).

#### Plagiopyxis glyphostoma

Bonnet, 1959

##### Distribution

**Vitosha Mt.** (Todorov 1993, Todorov and Golemansky 1995).

#### Plagiopyxis labiata

Penard, 1910

Centropyxia labiata Bartoš, 1947

##### Distribution

**Pirin Mt.** (Golemansky 1974); **Rila Mt.** (Golemansky and Todorov 1993).

#### Plagiopyxis minuta

Bonnet, 1959

##### Distribution

**Vitosha Mt.** (Todorov 1993, Todorov and Golemansky 1995).

#### Plagiopyxis oblonga

(Bonnet et Thomas, 1955)

##### Distribution

**Stara Planina Mt.** (new data).

#### Hyalospheniidae

(Schulze, 1877) Kosakyan et Lara, 2012

#### Hyalosphenia

(Stein, 1857) Schulze, 1877

#### Hyalosphenia elegans

(Leidy, 1874) Leidy, 1879

Difflugia (Catharia) elegans Leidy, 1874
Hyalosphenia turfacea Taránek, 1881

##### Distribution

**Vitosha Mt.** (Todorov 1993, Todorov and Golemansky 1995).

#### Hyalosphenia papilio

(Leidy, 1874) Leidy, 1875

Difflugia (Catharia) papilio Leidy, 1874

##### Distribution

**Pirin Mt.** (new data); **Rhodopes Mt.** (Golemansky 1968, Golemansky et al. 2006); **Rila Mt.** (Golemansky and Todorov 1993, Todorov and Golemansky 2000,Todorov 2005, new data); **Stara Planina Mt.** (new data); **Vitosha Mt.** (Pateff 1924, Golemansky 1965,Golemansky and Todorov 1985, Golemansky and Todorov 1990, Todorov 1993, Todorov and Golemansky 1995, new data).

#### Nebela

(Leidy, 1874) sensu Kosakyan, Lahr, Mulot, Meisterfeld, Mitchell and Lara, 2016

#### Nebela aliciae

Mitchell et Lara, 2013

##### Distribution

**Stara Planina Mt.** (new data); **Vitosha Mt.** (new data).

#### Nebela collaris

(Ehrenberg 1848) Leidy, 1879

Difflugia collaris Ehrenberg 1848
Diffluga cancellata Ehrenberg 1848
Difflugia reticulata Ehrenberg 1848
Difflugia carpio Ehrenberg 1854
Difflugia laxa Ehrenberg 1871
Difflugia cellulifera Ehrenberg 1874
Nebela numata Leidy 1874
Nebela bohemica Taranek 1882
Nebela sphagnophila (Steinecke) Van Oye 1933
Nebela tincta var. major Deflandre 1936
Nebela tincta f. stenostoma Jung 1936

##### Distribution

**Pirin Mt.** (Golemansky 1974, new data); **Rhodopes Mt.** (Pateff 1924, Golemansky 1968, Golemansky et al. 2006); **Rila Mt.** (Golemansky and Todorov 1993, Todorov and Golemansky 2000, Todorov 2005, new data); **Stara Planina Mt.** (new data); **Vitosha Mt.** (Pateff 1924, Golemansky 1965, Golemansky and Todorov 1985, Golemansky and Todorov 1990, Todorov 1993, Todorov and Golemansky 1995, Kosakyan et al. 2012; new data).

##### Notes

The species has been recorded as nominal species, as synonym *N. bohemica* (**Pirin Mt., Rhodopes Mt., Rila Mt., Vitosha Mt.**) and as infrasubspecific taxon Nebela tincta var. major (**Vitosha Mt.**).

#### Nebela flabellulum

Leidy, 1874

Difflugia (Nebela) flabellulum Leidy, 1874

##### Distribution

**Rhodopes Mt.** (Golemansky 1968, Golemansky et al. 2006); **Stara Planina Mt.** (new data).

#### Nebela guttata

Kosakyan et Lara, 2013

##### Distribution

**Pirin Mt.** (new data); **Rila Mt.** (new data); **Stara Planina Mt.** (new data); **Vitosha Mt.** (new data).

#### Nebela militaris

Penard, 1890

##### Distribution

**Pirin Mt.** (new data); **Rila Mt.** (Pateff 1924, new data); **Stara Planina Mt.** (new data); **Vitosha Mt.** (Pateff 1924, Golemansky 1965, Golemansky and Todorov 1990, Todorov 1993, Todorov and Golemansky 1995, new data).

#### Nebela pechorensis

Kosakyan et Mitchell, 2013

##### Distribution

**Pirin Mt.** (new data); **Rila Mt.** (new data); **Stara Planina Mt.** (new data); **Vitosha Mt.** (new data).

#### Nebela rotunda

Penard, 1890

Nebela tincta var. rotunda Penard, 1890

##### Distribution

**Vitosha Mt.** (new data).

#### Nebela tincta

(Leidy, 1879) Awerintzew, 1906

Hyalosphenia tincta Leidy, 1879
Nebela bursella Vejdovsky, 1882
Nebela minor Penard, 1902
Nebela parvula Cash, 1909

##### Distribution

**Pirin Mt.** (Golemansky 1974, new data); **Rhodopes Mt.** (Golemansky 1968, Golemansky et al. 2006); **Rila Mt.** (Pateff 1924, Golemansky and Todorov 1993, Todorov and Golemansky 2000, new data); **Stara Planina Mt.** (new data); **Vitosha Mt.** (Pateff 1924, Golemansky 1965, Golemansky and Todorov 1985, Golemansky and Todorov 1990, Todorov 1993, Todorov and Golemansky 1995, new data).

##### Notes

The species has been recorded both as nominal species and as synonyms *N. bursella* (**Rila Mt, Vitosha Mt.**), *N. minor* (**Rhodopes Mt., Rila Mt.**) and *N. parvula* (**Pirin Mt., Rhodopes Mt., Vitosha Mt.**).

#### Gibbocarina

Kosakyan, Lahr, Mulot, Meisterfeld, Mitchell and Lara, 2016

#### Gibbocarina galeata

(Penard, 1890) Kosakyan, Lahr, Mulot, Meisterfeld, Mitchell and Lara, 2016

Nebela galeata Penard, 1890

##### Distribution

**Pirin Mt.** (new data); **Rhodopes Mt.** (Pateff 1924, Golemansky 1968, Golemansky et al. 2006); **Rila Mt.** (Pateff 1924, Todorov and Golemansky 2000, Todorov 2005, new data); **Stara Planina Mt.** (new data); **Vitosha Mt.** (Pateff 1924, Golemansky 1965, Golemansky and Todorov 1985, Golemansky and Todorov 1990, Todorov 1993, Todorov and Golemansky 1995, new data).

##### Notes

So far, all records for the species have been as *N. galeata*.

#### Longinebela

Kosakyan, Lahr, Mulot, Meisterfeld, Mitchell and Lara, 2016

#### Longinebela ampulla

Todorov, Bankov and Ganeva, 2018

##### Distribution

**Stara Planina Mt.** (Todorov et al. 2018).

#### Longinebela golemanskyi

(Todorov, 2010) Kosakyan, Lahr, Mulot, Meisterfeld, Mitchell and Lara, 2016

Nebela golemanskyi Todorov, 2010

##### Distribution

**Vitosha Mt.** (Todorov 2010, new data).

##### Notes

The species has been recorded as *N. golemanskyi*.

#### Longinebela penardiana

(Deflandre, 1936) Kosakyan, Lahr, Mulot, Meisterfeld, Mitchell and Lara, 2016

Nebela penardiana Deflandre, 1936

##### Distribution

**Pirin Mt.** (Golemansky 1974, new data); **Rhodopes Mt.** (Golemansky 1968, Golemansky et al. 2006); **Rila Mt.** (Golemansky and Todorov 1993, Todorov and Golemansky 2000, Todorov 2005, new data); **Stara Planina Mt.** (new data); **Vitosha Mt.** (Golemansky 1965, Golemansky and Todorov 1985, Golemansky and Todorov 1990, Todorov 1993, Todorov and Golemansky 1995, Kosakyan et al. 2012, new data).

##### Notes

So far, all records for the species have been as *N. penardiana*.

#### Longinebela speciosa

(Deflandre, 1936) Kosakyan, Lahr, Mulot, Meisterfeld, Mitchell and Lara, 2016

Nebela speciosa Deflandre, 1936

##### Distribution

**Pirin Mt.** (new data); **Rhodopes Mt.** (Golemansky et al. 2006); **Rila Mt.** (Todorov and Golemansky 2000, new data); **Stara Planina Mt.** (new data); **Vitosha Mt.** (Golemansky 1965, Golemansky and Todorov 1985, Golemansky and Todorov 1990, Todorov 1993, Todorov and Golemansky 1995, Kosakyan et al. 2012, new data).

##### Notes

So far, all records for the species have been as *N. speciosa*.

#### Longinebela tubulosa

(Penard, 1902) Kosakyan, Lahr, Mulot, Meisterfeld, Mitchell and Lara, 2016

Nebela tubulosa Penard, 1902

##### Distribution

**Pirin Mt.** (new data); **Rila Mt.** (Todorov and Golemansky 2000, Todorov 2005, new data); **Stara Planina Mt.** (new data); **Vitosha Mt.** (Todorov 1993, Todorov and Golemansky 1995, Kosakyan et al. 2012, new data).

##### Notes

So far, all records for the species have been as *N. tubulosa*.

#### Padaungiella

Lara et Todorov, 2012

#### Padaungiella americana

(Taranek, 1882)

Nebela americana Taranek, 1882.

##### Distribution

**Rhodopes Mt.** (Pateff 1924, Golemansky et al. 2006); **Vitosha Mt.** (Pateff 1924, Golemansky 1965, Golemansky and Todorov 1990).

##### Notes

All records for the species have been as *N. americana* (**Rhodopes Mt., Vitosha Mt.**). This species is with doubtful identity since it is sharing overlapping characters with *P. lageniformis* and with *P. wailesi*.

#### Padaungiella lageniformis

(Penard, 1890) Lara et Todorov, 2012

Nebela lageniformis Penard, 1890

##### Distribution

**Pirin Mt.** (Golemansky 1974, new data); **Rhodopes Mt.** (Pateff 1924, Golemansky 1968, Golemansky et al. 2006); **Rila Mt.** (Pateff 1924, Golemansky and Todorov 1993, Todorov and Golemansky 2000, Todorov 2004, Todorov 2005, new data); **Stara Planina Mt.** (new data); **Vitosha Mt.** (Pateff 1924, Golemansky 1965, Golemansky and Todorov 1985, Golemansky and Todorov 1990, Todorov 1993, Todorov and Golemansky 1995, Kosakyan et al. 2012, new data).

##### Notes

All records for the species (except these in Kosakyan et al. 2012) have been as *N. lageniformis*.

#### Padaungiella nebeloides

(Gauthier-Lièvre and Thomas, 1958) Lara et Todorov, 2012

Difflugia nebeloides Gauthier-Lièvre and Thomas, 1958
Nebela nebeloides (Gauthier-Lièvre and Thomas, 1958) Todorov, Golemansky and Meisterfeld, 2010

##### Distribution

**Pirin Mt.** (new data); **Rila Mt.** (new data); **Stara Planina Mt.** (new data); **Vitosha Mt.** (Todorov et al. 2010, new data).

##### Notes

The species has been recorded by the synonymous name *N. nebeloides*. It should be noted that Mazei and Warren (2014) erroneously synonymised *D. nebeloides* with *D. linearis* (Penard, 1890) Gautier-Lièvre et Thomas, 1958, because they have not taken into account the fact that this species has recently been transferred from the genus *Difflugia* into the genus *Nebela* and subsequently in the newly described genus *Padaungiella* (Todorov et al. 2010, Kosakyan et al. 2012).

#### Padaungiella tubulata

(Brown, 1911) Lara et Todorov, 2012

Nebela tubulata Brown, 1911

##### Distribution

**Pirin Mt.** (new data); **Rhodopes Mt.** (Golemansky et al. 2006); **Rila Mt.** (Todorov 2005, new data); **Stara Planina Mt.** (new data); **Vitosha Mt.** (Golemansky 1965, Golemansky and Todorov 1990, Todorov 1993, Todorov and Golemansky 1995, new data).

##### Notes

So far, all records for the species have been as *N. tubulata*.

#### Padaungiella wailesi

(Deflandre, 1936) Lara et Todorov, 2012

Nebela wailesi Deflandre, 1936

##### Distribution

**Rhodopes Mt.** (Golemansky et al. 2006).

##### Notes

The species has been recorded by the synonymous name *N. wailesi*.

#### Planocarina

Kosakyan, Lahr, Mulot, Meisterfeld, Mitchell and Lara, 2016

#### Planocarina carinata

(Archer, 1867) Kosakyan, Lahr, Mulot, Meisterfeld, Mitchell and Lara, 2016

Nebela carinata (Archer, 1867) Leidy, 1879

##### Distribution

**Rila Mt.** (Golemansky and Todorov 1993); **Stara Planina Mt.** (new data); **Vitosha Mt.** (Golemansky and Todorov 1985, Golemansky and Todorov 1990, Todorov 1993, Todorov and Golemansky 1995).

##### Notes

So far, all records for the species have been as *N. carinata*.

#### Quadrulella

(Cockerell, 1909) sensu Kosakyan, Lahr, Mulot, Meisterfeld, Mitchell and Lara, 2016

#### Quadrulella longicollis

(Taranek, 1882)

Quadrulella symmetrica var. longicollis Taranek, 1882

##### Distribution

**Pirin Mt.** (new data); **Rhodopes Mt.** (Golemansky et al. 2006); **Rila Mt.** (Todorov and Golemansky 2000, Todorov 2004, Todorov 2005, new data); **Stara Planina Mt.** (new data); **Vitosha Mt.** (Golemansky 1965, Golemansky and Todorov 1985, Golemansky and Todorov 1990, Todorov 1993, Todorov and Golemansky 1995, Kosakyan et al. 2012, new data).

##### Notes

So far, all records for the species (except this one of Kosakyan et al. 2012) have been as the infrasubspecific taxon Q. symmetrica var. longicollis (**Rhodopes Mt., Rila Mt., Vitosha Mt.**).

#### Quadrulella symmetrica

(Wallich, 1864) Cockerell, 1909

Difflugia proteiformis var. symmetrica Wallich, 1863
Difflugia pyriformis var. symmetrica Wallich, 1864
Difflugia symmetrica Wallich, 1864
Assulina assulata Ehrenberg, 1871
Assulina leptolepis Ehrenberg, 1871
Difflugia assulata Ehrenberg, 1871
Difflugia carolensis Ehrenberg, 1871
Quadrula symmetrica Schulze, 1875
Nebela (Quadrulella) symmetrica Deflandre, 1936

##### Distribution

**Pirin Mt.** (Golemansky 1974, new data); **Rhodopes Mt.** (Pateff 1924, Golemansky et al. 2006); **Rila Mt.** (Pateff 1924, Golemansky and Todorov 1993, Todorov and Golemansky 2000, Todorov 2004,Todorov 2005, new data); **Stara Planina Mt.** (new data); **Vitosha Mt.** (Pateff 1924, Golemansky 1965, Golemansky and Todorov 1985, Golemansky and Todorov 1990, Todorov 1993, Todorov and Golemansky 1995, Kosakyan et al. 2012, new data).

##### Notes

Besides the nominal species, the infrasubspecific taxon Quadrulella symmetrica var. irregularis Wailes et Penard, 1911 has also been recorded (**Rhodopes Mt., Rila Mt., Vitosha Mt.**).

#### Quadrulella tubulata

Gauthier-Lièvre, 1953

##### Distribution

**Pirin Mt.** (Golemansky 1974).

#### Quadrulella variabilis

Kosakyan, Lahr, Mulot, Meisterfeld, Mitchell and Lara, 2016

##### Distribution

**Pirin Mt.** (new data); **Rila Mt.** (new data); **Stara Planina Mt.** (new data); **Vitosha Mt.** (new data).

#### Phryganellina

Bovee, 1985

#### Phryganellidae

Jung, 1942

#### Phryganella

Penard, 1902

#### Phryganella acropodia

(Hertwig and Lesser, 1874) Hopkinson, 1909

Difflugia acropodia Hertwig and Lesser, 1874

##### Distribution

**Pirin Mt.** (new data); **Rhodopes Mt.** (Golemansky et al. 2006); **Rila Mt.** (Todorov 2004, Todorov 2005, new data); **Stara Planina Mt.** (new data); **Vitosha Mt.** (Golemansky and Todorov 1985, Golemansky and Todorov 1990, Todorov 1993, Todorov and Golemansky 1995, new data).

#### Phryganella hemisphaerica

(Penard, 1890) Penard, 1902

Pseudodifflugia hemisphaerica Penard, 1890
Difflugia globulosa Leidy, 1879 (in part)

##### Distribution

**Pirin Mt.** (Golemansky 1974, new data); **Rhodopes Mt.** (Golemansky 1968, Golemansky et al. 2006); **Rila Mt.** (Todorov and Golemansky 2000, Todorov 2004,Todorov 2005, new data); **Stara Planina Mt.** (new data); **Vitosha Mt.** (new data)

#### Phryganella nidulus

Penard, 1902

Difflugia globulosa Leidy, 1879 (in part)

##### Distribution

**Pirin Mt.** (new data); **Rhodopes Mt.** (Pateff 1924, Golemansky et al. 2006); **Rila Mt.** (Todorov 2004, Todorov 2005, new data); **Vitosha Mt.** (Pateff 1924).

#### Phryganella paradoxa

Penard, 1902

##### Distribution

**Rhodopes Mt.** (Golemansky et al. 2006); **Stara Planina Mt.** (new data); **Vitosha Mt.** (Golemansky and Todorov 1985, Golemansky and Todorov 1990, Todorov 1993, Todorov and Golemansky 1995, new data).

#### Cryptodifflugiidae

Jung, 1942

#### Cryptodifflugia

Penard, 1890

#### Cryptodifflugia compressa

Penard, 1902

##### Distribution

**Pirin Mt.** (new data); **Rhodopes Mt.** (Golemansky 1968, Golemansky et al. 2006); **Rila Mt.** (new data); **Stara Planina Mt.** (new data); **Vitosha Mt.** (Golemansky 1965, Golemansky and Todorov 1990, new data).

#### Cryptodifflugia oviformis

Penard, 1890

Difflugiella oviformis Bonnet and Thomas, 1955

##### Distribution

**Pirin Mt.** (new data); **Rhodopes Mt.** (Golemansky 1968, Golemansky et al. 2006); **Rila Mt.** (Golemansky and Todorov 1993, Todorov and Golemansky 2000, Todorov 2004, new data); **Stara Planina Mt.** (new data); **Vitosha Mt.** (Pateff 1928, Golemansky and Todorov 1985, Golemansky and Todorov 1990, Todorov 1993, Todorov and Golemansky 1995, new data).

##### Notes

The species has been recorded both as nominal species and as synonym *D. oviformis* (**Rila Mt., Vitosha Mt.**), as well as infrasubspecific taxon Difflugiella oviformis var. fusca Bonnet and Thomas, 1955 (**Rhodopes Mt., Rila Mt., Vitosha Mt.**).

#### Wailesella

Deflandre, 1928

#### Wailesella eboracensis

(Wailes and Penard, 1911) Deflandre, 1928

Cryptodifflugia eboracensis Wailes and Penard, 1911

##### Distribution

**Pirin Mt.** (new data); **Rhodopes Mt.** (Golemansky et al. 2006); **Rila Mt.** (Golemansky and Todorov 1993, Todorov and Golemansky 2000, new data); **Stara Planina Mt.** (new data); **Vitosha Mt.** (Pateff 1928, Golemansky 1965, Golemansky and Todorov 1985, Golemansky and Todorov 1990, Todorov 1993, Todorov and Golemansky 1995, new data).

##### Notes

The species has been recorded both as nominal species and as synonym *C. eboracensis* (**Vitosha Mt.**).

#### Arcellinida

#### Argynnia

Vucetich, 1974

#### Argynnia bipes

(Carter, 1870) Murray, 1870

Difflugia bipes Carter, 1870
Nebela bipes Murray, 1870
Nebela bicornis West, 1905

##### Distribution

**Rhodopes Mt.** (Pateff 1928, Golemansky et al. 2006); **Vitosha Mt.** (Pateff 1928, Golemansky and Todorov 1990).

##### Notes

The species has been recorded as synonym *N. bipes* (**Rhodopes Mt., Vitosha Mt.**).

#### Argynnia dentistoma

(Penard, 1890)

Nebela dentistoma Penard, 1890
Nebela crenulata Penard, 1893
Nebela collaris Leidy, 1879 (in part)

##### Distribution

**Pirin Mt.** (Golemansky 1974, new data); **Rhodopes Mt.** (Pateff 1924, Golemansky 1968, Golemansky et al. 2006); **Rila Mt.** (Pateff 1924, Golemansky and Todorov 1993, Todorov and Golemansky 2000, Todorov 2005, new data); **Stara Planina Mt.** (new data); **Vitosha Mt.** (Pateff 1924, Golemansky 1965, Golemansky and Todorov 1985, Golemansky and Todorov 1990, Todorov 1993, Todorov and Golemansky 1995, new data).

##### Notes

So far, all records for the species have been as synonyms *N. dentistoma* (**Pirin Mt., Rhodopes Mt., Rila Mt., Vitosha Mt.**), *N. crenulata* (**Rhodopes Mt., Rila Mt., Vitosha Mt.**), as well as infrasubspecific taxa Nebela dentistoma var. oblonga Gauthier-Lièvre, 1853 (**Rila Mt.**).

#### Argynnia vitraea

(Penard, 1899)

Nebela vitraea Penard, 1899

##### Distribution

**Pirin Mt.** (new data); **Rhodopes Mt.** (Golemansky et al. 2006); **Rila Mt.** (Todorov and Golemansky 2000, Todorov 2004, Todorov 2005, new data); **Stara Planina Mt.** (new data); **Vitosha Mt.** (Golemansky and Todorov 1985, Golemansky and Todorov 1990, Todorov 1993, Todorov and Golemansky 1995, new data).

##### Notes

The species has been recorded as synonym *N. vitraea* (**Rhodopes Mt., Rila Mt., Vitosha Mt.**).

#### Awerintzewia

Schouteden, 1906

#### Awerintzewia cyclostoma

(Penard, 1902) Schouteden, 1906

Heleopera cyclostoma Penard, 1902

##### Distribution

**Rhodopes Mt.** (Pateff 1924, Golemansky et al. 2006).

##### Notes

The species has been recorded both as nominal species and as synonym *H. cyclostoma* (**Rhodopes Mt.**).

#### Heleopera

Leidy, 1879

#### Heleopera petricola

Leidy, 1879

##### Distribution

**Pirin Mt.** (new data); **Rhodopes Mt.** (Pateff 1924, Golemansky et al. 2006); **Rila Mt.** (Golemansky and Todorov 1993, Todorov 2005, new data); **Stara Planina Mt.** (new data); **Vitosha Mt.** (Pateff 1924, Golemansky 1965, Golemansky and Todorov 1985, Golemansky and Todorov 1990, Todorov 1993, Todorov and Golemansky 1995, new data).

##### Notes

Besides the nominal species, two infrasubspecific taxa Heleopera petricola var. amethystea Penard, 1899 (**Rila Mt.**) and Heleopera petricola var. major Deflandre, 1928 (**Vitosha Mt.**) have also been recorded.

#### Heleopera rosea

Penard, 1890

##### Distribution

**Pirin Mt.** (Golemansky 1974, new data); **Rhodopes Mt.** (Golemansky 1968, Golemansky et al. 2006); **Rila Mt.** (Golemansky and Todorov 1993, Todorov and Golemansky 2000, Todorov 2005, new data); **Stara Planina Mt.** (new data); **Vitosha Mt.** (Pateff 1924, Golemansky 1965, Golemansky and Todorov 1985, Golemansky and Todorov 1990, Todorov 1993, Todorov and Golemansky 1995, new data).

#### Heleopera sphagni

(Leidy, 1874)

Difflugia (Nebela) sphagni Leidy, 1874
Nebela sphagni Leidy, 1876
Nebela picta Leidy, 1879

##### Distribution

**Rhodopes Mt.** (Golemansky 1968, Golemansky et al. 2006); **Rila Mt.** (Golemansky and Todorov 1993).

#### Heleopera sylvatica

Penard, 1890

##### Distribution

**Rhodopes Mt.** (Golemansky et al. 2006); **Rila Mt.** (Golemansky and Todorov 1993, Todorov 2005, new data); **Vitosha Mt.** (Golemansky 1965, Golemansky and Todorov 1990).

#### Lesquereusia

Schlumberger, 1845

#### Lesquereusia epistomium

Penard, 1902

Lecquereusia jurassica var. epistomium Penard, 1893
Lecquereusia epistomium Penard, 1902

##### Distribution

**Pirin Mt.** (new data); **Rhodopes Mt.** (Pateff 1924, Golemansky 1968,Golemansky et al. 2006); **Rila Mt.** (Todorov and Golemansky 2000, Todorov 2005, new data); **Stara Planina Mt.** (new data); **Vitosha Mt.** (Pateff 1924, Golemansky 1965, Golemansky and Todorov 1985, Golemansky and Todorov 1990, Todorov 1993, Todorov and Golemansky 1995, new data).

#### Lesquereusia gibbosa

Thomas and Gauthier-Lièvre, 1859

##### Distribution

**Pirin Mt.** (new data); **Rhodopes Mt.** (Golemansky 1968, Golemansky et al. 2006); **Rila Mt.** (Todorov 2005, new data); **Stara Planina Mt.** (new data); **Vitosha Mt.** (Golemansky and Todorov 1990, Todorov 1993, Todorov and Golemansky 1995, new data).

#### Lesquereusia modesta

Rhumbler, 1896

Difflugia spiralis Leidy, 1879 (in part)

##### Distribution

**Pirin Mt.** (new data); **Rhodopes Mt.** (Golemansky 1968, Golemansky et al. 2006); **Rila Mt.** (Todorov 2004, Todorov 2005, new data); **Stara Planina Mt.** (new data); **Vitosha Mt.** (Golemansky 1965, Golemansky and Todorov 1990, Todorov 1993, Todorov and Golemansky 1995, new data).

#### Lesquereusia spiralis

(Ehrenberg, 1840) Bütschli, 1880

Difflugia spiralis Ehrenberg, 1840
Lecquereusia jurassica Schlumberger, 1845
Difflugia helix Cohn, 1853

##### Distribution

**Pirin Mt.** (new data); **Rhodopes Mt.** (Pateff 1924, Golemansky 1968, Golemansky et al. 2006); **Rila Mt.** (Todorov and Golemansky 2000, Todorov 2004, Todorov 2005, new data); **Stara Planina Mt.** (new data); **Vitosha Mt.** (Pateff 1924, Golemansky 1965, Golemansky and Todorov 1985, Golemansky and Todorov 1990, Todorov 1993, Todorov and Golemansky 1995, new data).

#### Pyxidicula

Ehrenberg, 1838

#### Pyxidicula patens

(Claparede and Lachmann, 1858)

Arcella patens Claparede and Lachmann, 1858

##### Distribution

**Rhodopes Mt.** (Golemansky 1968, Golemansky et al. 2006).

#### Rhizaria

Cavalier-Smith, 2002

#### Stramenopiles

Patterson, 1989, emend. Adl et al., 2005

#### Labyrinthomycetes

Dick, 2001

#### Amphitremida

(Poch, 1913) Gomma, Mitchell and Lara, 2013

#### Amphitremidae

Poch, 1913

#### Archerella

Loeblich and Tappan, 1961

#### Archerella flavum

(Archer, 1877) Loeblich and Tappan, 1961

Ditrema flavum Archer, 1877
Amphitrema flavum Archer, 1877

##### Distribution

**Rhodopes Mt.** (Golemansky 1968, Golemansky et al. 2006); **Rila Mt.** (new data); **Stara Planina Mt.** (new data); **Vitosha Mt.** (Pateff 1924, Golemansky 1965, Golemansky and Todorov 1985, Golemansky and Todorov 1990, Todorov 1993, Todorov and Golemansky 1995, new data).

##### Notes

The species has been recorded as synonym *A. flavum* (**Rhodopes Mt., Vitosha Mt.**).

#### Cercozoa

Cavalier-Smith, 1998, emend. Adl et al., 2005

#### Filosa

Cavalier-Smith, 2003

#### Silicofilosea

Adl et al., 2005, emend. Adl et al., 2012

#### Euglyphida

Copeland, 1956, emend. Cavalier-Smith, 1997

#### Euglyphidae

Wallich, 1864

#### Euglypha

Dujardin, 1841

#### Euglypha acanthophora

(Ehrenberg, 1841) Perty, 1849

Difflugia acanthophora Ehrenberg, 1841
Euglypha alveolata Dujardin, 1841 (in part)
Euglypha setigera Perty, 1852 (in part)
Difflugia setigera Ehrenberg, 1871
Euglypha acanthophora *Difflugia Setigerella acanthophora* Ehrenberg, 1871
Euglypha brachiata Penard, 1902 (non Leidy, 1879)
Euglypha alveolata var. gracilis Taránek, 1881 (in part)
Euglypha armata Wailes and Penard, 1911
Euglypha australica Playfair, 1918 (in part)

##### Distribution

**Pirin Mt.** (Golemansky 1974, new data); **Rhodopes Mt.** (Pateff 1924, Golemansky 1968, Golemansky et al. 2006); **Rila Mt.** (Todorov and Golemansky 2000, Todorov 2004, Todorov 2005, new data); **Stara Planina Mt.** (new data); **Vitosha Mt.** (Pateff 1924, Golemansky 1965, Golemansky and Todorov 1985, Golemansky and Todorov 1990, Todorov 1993, Todorov and Golemansky 1995, new data).

##### Notes

Pateff (1924) and Golemansky et al. (2006) erroneously recorded the species *E. brachiata* (**Rhodopes Mt., Vitosha Mt.**), because the description of the found individuals fully corresponds to *E. acanthophora*.

#### Euglypha aspera

Penard, 1899

##### Distribution

**Pirin Mt.** (new data); **Rhodopes Mt.** (Pateff 1924, Golemansky et al. 2006); **Rila Mt.** (Todorov 2004, new data).

#### Euglypha bryophila

Brown, 1911

Euglypha Vejdovsky, 1882
Euglypha cristata Penard, 1890 (in part)

##### Distribution

**Pirin Mt.** (new data); **Rhodopes Mt.** (Golemansky et al. 2006); **Rila Mt.** (Golemansky and Todorov 1993, Todorov and Golemansky 2000, new data); **Stara Planina Mt.** (new data); **Vitosha Mt.** (Golemansky 1965, Golemansky and Todorov 1990, new data).

#### Euglypha ciliata

(Ehrenberg, 1848), Leidy, 1878

Difflugia ciliata Ehrenberg, 1848
Euglypha setigera Perty, 1852 (in part)
Difflugia pilosa Ehrenberg, 1871
Euglypha ciliata *DifflugiaSetigerellaciliata* Ehrenberg, 1871
Euglypha ciliata *Difflugia Setigerella pilosa* Ehrenberg, 1871

##### Distribution

**Pirin Mt.** (new data); **Rhodopes Mt.** (Pateff 1924, Golemansky 1968, Golemansky et al. 2006); **Rila Mt.** (Golemansky and Todorov 1993, Todorov and Golemansky 2000, Todorov 2004, Todorov 2005, new data); **Stara Planina Mt.** (new data); **Vitosha Mt.** (Pateff 1924, Golemansky and Todorov 1985, Golemansky and Todorov 1990, Todorov 1993, Todorov and Golemansky 1995, new data).

##### Notes

The species has been recorded both as nominal species and as infrasubspecific taxon E. ciliata f. glabra Wailes, 1915 (**Rhodopes Mt., Rila Mt.**).

#### Euglypha compressa

Carter, 1864

Euglypha ampullacea Hertwig and Lesser, 1874
Euglypha ciliata Leidy, 1879 (in part)
Euglypha Vejdovsky, 1882 (in part)
Euglypha compressa ? *Euglypha zonata* Maggi, 1888

##### Distribution

**Pirin Mt.** (new data); **Rhodopes Mt.** (Golemansky et al. 2006); **Rila Mt.** (Todorov 2005, new data); **Stara Planina Mt.** (new data); **Vitosha Mt.** (Golemansky 1965, Golemansky and Todorov 1985, Golemansky and Todorov 1990, Todorov 1993, Todorov and Golemansky 1995, new data).

##### Notes

The species has been recorded both as nominal species and as infrasubspecific taxon E. compressa f. glabra Wailes, 1915 (**Rhodopes Mt.**).

#### Euglypha crenulata

Wailes, 1912

##### Distribution

**Vitosha Mt.** (Golemansky and Todorov 1985, Golemansky and Todorov 1990, Todorov 1993, Todorov and Golemansky 1995).

##### Notes

The species has been recorded as infrasubspecific taxon E. crenulata var. minor Wailes, 1912.

#### Euglypha cristata

Leidy, 1874

##### Distribution

**Pirin Mt.** (new data); **Rhodopes Mt.** (Golemansky 1968, Golemansky et al. 2006); **Rila Mt.** (Pateff 1924, Golemansky and Todorov 1993, Todorov and Golemansky 2000, Todorov 2005, new data); **Stara Planina Mt.** (new data); **Vitosha Mt.** (Pateff 1924, Golemansky 1965, Golemansky and Todorov 1985, Golemansky and Todorov 1990, Todorov 1993, Todorov and Golemansky 1995, new data).

#### Euglypha denticulata

Brown, 1912

##### Distribution

**Pirin Mt.** (new data); **Rhodopes Mt.** (Golemansky et al. 2006); **Rila Mt.** (Todorov and Golemansky 2000, Todorov 2005, new data); **Stara Planina Mt.** (new data); **Vitosha Mt.** (new data).

#### Euglypha filifera

Penard, 1890

Euglypha setigera Perty, 1852 (in part)
Euglypha ciliata Leidy, 1879 (in part)
Euglypha longispina Taránek, 1881

##### Distribution

**Pirin Mt.** (Golemansky 1974, new data); **Rhodopes Mt.** (Golemansky et al. 2006); **Rila Mt.** (Todorov and Golemansky 2000, Todorov 2004, Todorov 2005, new data); **Stara Planina Mt.** (new data); **Vitosha Mt.** (Golemansky 1965, Golemansky and Todorov 1985, Golemansky and Todorov 1990,Todorov 1993, Todorov and Golemansky 1995, new data).

#### Euglypha laevis

(Ehrenberg, 1845) Perty, 1849

Difflugia laevis Ehrenberg, 1845
Euglypha alveolata Leidy, 1879 (in part)
Euglypha Vejdovsky, 1882

##### Distribution

**Pirin Mt.** (Golemansky 1974, new data); **Rhodopes Mt.** (Golemansky 1968, Golemansky et al. 2006); **Rila Mt.** (Pateff 1924,Golemansky and Todorov 1993, Todorov and Golemansky 2000, Todorov 2005, new data); **Stara Planina Mt.** (new data); **Vitosha Mt.** (Golemansky 1965, Golemansky and Todorov 1985, Golemansky and Todorov 1990, Todorov 1993, Todorov and Golemansky 1995, new data).

#### Euglypha rotunda

Wailes and Penard, 1911

##### Distribution

**Pirin Mt.** (new data); **Rhodopes Mt.** (Golemansky 1968, Golemansky et al. 2006); **Rila Mt.** (Golemansky and Todorov 1993, Todorov and Golemansky 2000, Todorov 2005, new data); **Stara Planina Mt.** (new data); **Vitosha Mt.** (Golemansky 1965, Golemansky and Todorov 1990, Todorov 1993, Todorov and Golemansky 1995, new data).

#### Euglypha strigosa

(Ehrenberg, 1871) Leidy, 1878

Difflugia strigosa Ehrenberg, 1871
Euglypha strigosa *DifflugiaSetigerellastrigosa* Ehrenberg, 1871
Euglypha ciliata var. strigosa Leidy, 1879 (in part)
Euglypha heterospina Penard, 1890

##### Distribution

**Pirin Mt.** (Golemansky 1974, new data); **Rhodopes Mt.** (Golemansky 1968, Golemansky et al. 2006); **Rila Mt.** (Golemansky and Todorov 1993, Todorov and Golemansky 2000, Todorov 2005, new data); **Stara Planina Mt.** (new data); **Vitosha Mt.** (Pateff 1928, Golemansky 1965, Golemansky and Todorov 1985, Golemansky and Todorov 1990,Todorov 1993, Todorov and Golemansky 1995, new data).

##### Notes

Besides the nominal species, two infrasubspecific taxa Euglypha strigosa f. glabra Wailes and Penard, 1911 (**Rila Mt.**) and Euglypha strigosa f. heterospina Wailes and Penard, 1911 (**Rhodopes Mt., Rila Mt., Vitosha Mt.**) have also been recorded.

#### Euglypha tuberculata

Dujardin, 1841

Difflugia areolata Ehrenberg, 1841
Euglypha alveolata Dujardin, 1841 (in part)
Euglypha tuberculosa Dujardin, 1841
Difflugia alveolata Pritchard, 1861
Euglypha pusilla Entz, 1877
Euglypha Vejdovsky, 1882

##### Distribution

**Pirin Mt.** (Golemansky 1974, new data); **Rhodopes Mt.** (Pateff 1924, Golemansky et al. 2006); **Rila Mt.** (Golemansky and Todorov 1993, Todorov and Golemansky 2000, Todorov 2005, new data); **Stara Planina Mt.** (new data); **Vitosha Mt.** (Pateff 1924, Golemansky 1965, Golemansky and Todorov 1985, Golemansky and Todorov 1990, Todorov 1993, Todorov and Golemansky 1995, new data).

##### Notes

The species has been recorded both as nominal species and as synonym *E. alveolata* (**Rhodopes Mt., Vitosha Mt.**).

#### Trinematidae

Hoogenraad and De Groot 1940, emend Adl et al. 2012

#### Trinema

Dujardin, 1841

#### Trinema complanatum

Penard, 1890

Trinema complanatum ? *Arcella nidus-pendulus* Ehrenberg, 1841
Trinema acinus Leidy, 1879 (in part)

##### Distribution

**Pirin Mt.** (new data); **Rhodopes Mt.** (Pateff 1924, Golemansky 1968, Golemansky et al. 2006); **Rila Mt.** (Golemansky and Todorov 1993, Todorov and Golemansky 2000, Todorov 2005, new data); **Stara Planina Mt.** (new data); **Vitosha Mt.** (Pateff 1924, Golemansky 1965, Golemansky and Todorov 1985, Golemansky and Todorov 1990, Todorov 1993, Todorov and Golemansky 1995, new data).

##### Notes

Besides the nominal species, two infrasubspecific taxa Trinema complanatum var. aerophila (Decloître, 1950) Bonnet and Thomas, 1960 (**Vitosha Mt.**) and Trinema complanatum var. globulosa Chardez, 1959 (**Rhodopes Mt., Vitosha Mt.**) have also been recorded.

#### Trinema enchelys

(Ehrenberg, 1838) Leidy, 1878

Difflugia enchelys Ehrenberg, 1838 (in part)
Trinema acinus Dujardin, 1841
Arcella enchelys Ehrenberg, 1844
Euglypha pleurostoma Carter, 1857
Euglypha enchelys Wallich, 1864
Trinema (Difflugia) encheli Crevier, 1870

##### Distribution

**Pirin Mt.** (Golemansky 1974, new data); **Rhodopes Mt.** (Pateff 1924, Golemansky 1968, Golemansky et al. 2006); **Rila Mt.** (Pateff 1924, Golemansky and Todorov 1993, Todorov and Golemansky 2000, Todorov 2005, new data); **Stara Planina Mt.** (new data); **Vitosha Mt.** (Pateff 1924, Golemansky 1965, Golemansky and Todorov 1985, Golemansky and Todorov 1990, Todorov 1993, Todorov and Golemansky 1995, new data).

#### Trinema galeata

(Penard, 1890) Jung, 1942

Trinema enchelys var. galeata Penard, 1890

##### Distribution

**Rila Mt.** (Golemansky and Todorov 1993).

#### Trinema grandis

(Chardez, 1960) Golemansky, 1963

Trinema enchelys var. grandis Chardez, 1960

##### Distribution

**Rila Mt.** (Golemansky and Todorov 1993, new data).

#### Trinema lineare

Penard, 1890

Difflugia enchelys Ehrenberg, 1838 (in part)
Arcella hyalina Ehrenberg, 1841
Arcella enchelys Ehrenberg, 1854
Trinema acinus Leidy, 1879 (in part)
Trinema enchelys forma *β* Awerintzew, 1906

##### Distribution

**Pirin Mt.** (Golemansky 1974, new data); **Rhodopes Mt.** (Golemansky 1968, Golemansky et al. 2006); **Rila Mt.** (Golemansky and Todorov 1993, Todorov and Golemansky 2000, Todorov 2004, Todorov 2005, new data); **Stara Planina Mt.** (new data); **Vitosha Mt.** (Golemansky 1965, Golemansky and Todorov 1985, Golemansky and Todorov 1990, Todorov 1993, Todorov and Golemansky 1995, new data).

#### Corythion

Taranek, 1881

#### Corythion delamarei

Bonnet and Thomas, 1960

##### Distribution

**Vitosha Mt.** (Todorov 1993, Todorov and Golemansky 1995).

#### Corythion dubium

Taránek, 1881

Arcella constricta Ehrenberg, 1841 (in part)
Arcella disphaera Ehrenberg, 1841 (in part)
Trinema acinus Leidy, 1879 (in part)
Trinema constricta Certes, 1889

##### Distribution

**Pirin Mt.** (Golemansky 1974, new data); **Rhodopes Mt.** (Golemansky 1968, Golemansky et al. 2006); **Rila Mt.** (Pateff 1924, Golemansky and Todorov 1993, Todorov and Golemansky 2000, Todorov 2004, Todorov 2005, new data); **Stara Planina Mt.** (new data); **Vitosha Mt.** (Pateff 1924, Golemansky 1965, Golemansky and Todorov 1985, Golemansky and Todorov 1990, Todorov 1993, Todorov and Golemansky 1995, new data).

##### Notes

Besides the nominal species, two infrasubspecific taxa Corythion dubium var. aerophila Decloître, 1950 (**Vitosha Mt.**) and Corythion dubium var. orbicularis Penard, 1910 (**Vitosha Mt.**) have also been recorded.

#### Playfairina

Thomas, 1961

#### Playfairina valkanovi

Golemansky, 1966

##### Distribution

**Pirin Mt.** (new data); **Rhodopes Mt.** (Golemansky et al. 2006); **Rila Mt.** (Golemansky and Todorov 1993, Todorov and Golemansky 2000, Todorov 2005, new data); **Stara Planina Mt.** (new data); **Vitosha Mt.** (Golemansky 1966, Golemansky and Todorov 1990, Todorov 1993, Todorov and Golemansky 1995, Golemansky and Todorov 2006,new data).

#### Sphenoderiidae

Chatelain, Meisterfeld, Roussel-Delif and Lara, 2013

#### Sphenoderia

Schlumberger, 1845

#### Sphenoderia fissirostris

Penard, 1890

##### Distribution

**Pirin Mt.** (Golemansky 1974, new data); **Rhodopes Mt.** (Golemansky 1968, Golemansky et al. 2006); **Rila Mt.** (Pateff 1924, Golemansky and Todorov 1993, Todorov and Golemansky 2000, Todorov 2004, Todorov 2005, new data); **Stara Planina Mt.** (new data); **Vitosha Mt.** (Pateff 1924, Golemansky 1965, Golemansky and Todorov 1985, Golemansky and Todorov 1990, Todorov 1993,Todorov and Golemansky 1995, new data).

#### Sphenoderia lenta

Schlumberger, 1845

##### Distribution

**Pirin Mt.** (Golemansky 1974, new data); **Rhodopes Mt.** (Pateff 1924, Golemansky et al. 2006); **Rila Mt.** (Pateff 1924, Todorov and Golemansky 2000, Todorov 2004, Todorov 2005, new data); **Stara Planina Mt.** (new data); **Vitosha Mt.** (Pateff 1924, Golemansky 1965, Golemansky and Todorov 1985, Golemansky and Todorov 1990, Todorov 1993, Todorov and Golemansky 1995, new data).

#### Sphenoderia minuta

Deflandre, 1931

##### Distribution

**Pirin Mt.** (new data); **Rhodopes Mt.** (Golemansky 1968, Golemansky et al. 2006); **Rila Mt.** (Golemansky and Todorov 1993, new data); **Stara Planina Mt.** (new data); **Vitosha Mt.** (Golemansky 1965, Golemansky and Todorov 1990, Todorov 1993, Todorov and Golemansky 1995, new data).

#### Sphenoderia ovoidea

Jung, 1942

##### Distribution

**Stara Planina Mt.** (new data); **Vitosha Mt.** (new data).

#### Sphenoderia splendida

(Playfair, 1917) Deflandre, 1931

Sphenoderia fissirostris var. splendida Playfair, 1917

##### Distribution

**Pirin Mt.** (new data); **Rila Mt.** (new data); **Stara Planina Mt.** (new data); **Vitosha Mt.** (new data).

#### Trachelocorythion

Bonnet, 1979

#### Trachelocorythion pulchellum

(Penard, 1890) Bonnet, 1979

Trachelocorythion pulchellum ? *Euglypha minima* Perty, 1852
Corythion pulchellum Penard, 1890
Chorythion pulchellum Awerintzew, 1907
Trachelocorythion pulchellum ? *Hyalina neta* Jung, 1942

##### Distribution

**Pirin Mt.** (new data); **Rhodopes Mt.** (Golemansky et al. 2006); **Rila Mt.** (Todorov and Golemansky 2000, new data); **Stara Planina Mt.** (new data); **Vitosha Mt.** (new data).

#### Assulinidae

Lara et al., 2007

#### Assulina

Leidy, 1879

#### Assulina muscorum

Greeff, 1888

Assulina seminulum Leidy, 1879 (in part)
Assulina minor Penard, 1890

##### Distribution

**Pirin Mt.** (new data); **Rhodopes Mt.** (Golemansky 1968, Golemansky et al. 2006); **Rila Mt.** (Golemansky and Todorov 1993, Todorov and Golemansky 2000, Todorov 2005, new data); **Stara Planina Mt.** (new data); **Vitosha Mt.** (Golemansky 1965, Golemansky and Todorov 1990, Todorov 1993, Todorov and Golemansky 1995, new data).

#### Assulina seminulum

(Ehrenberg, 1848) Leidy, 1879

Difflugia seminulum Ehrenberg, 1848
Assulina seminulum *DifflugiaAssulina seminulum* Ehrenberg, 1871
Difflugia semen Ehrenberg, 1871
Euglypha brunnea Leidy, 1874
Euglypha seminulum Leidy, 1878

##### Distribution

**Rhodopes Mt.** (Golemansky 1968, Golemansky et al. 2006); **Rila Mt.** (Pateff 1924, Golemansky and Todorov 1993, new data); **Stara Planina Mt.** (new data); **Vitosha Mt.** (Pateff 1924, Golemansky 1965, Golemansky and Todorov 1990, new data).

#### Placocista

Leidy, 1879

#### Placocista glabra

Penard, 1906

##### Distribution

**Pirin Mt.** (Golemansky 1974).

##### Notes

The species has been recorded as infrasubspecific taxon Placocista glabra var. minima Decloître, 1955 (**Pirin Mt.**).

#### Placocista spinosa

(Carter, 1865) Leidy, 1879

Euglypha spinosa Carter, 1865

##### Distribution

**Rila Mt.** (Golemansky and Todorov 1993).

#### Cyphoderiidae

de Saedeleer, 1934

#### Cyphoderia

Schlumberger, 1845

#### Cyphoderia amphoralis

(Wailes and Penard, 1911)

Cyphoderia trochus var. amphoralis Wailes and Penard, 1911

##### Distribution

**Pirin Mt.** (new data); **Rhodopes Mt.** (Todorov et al. 2009); **Rila Mt.** (Heger et al. 2010, new data); **Stara Planina Mt.** (new data); **Vitosha Mt.** (new data).

#### Cyphoderia ampulla

(Ehrenberg, 1840) Leidy, 1878

Difflugia ampulla Ehrenberg, 1840
Difflugia lagena Ehrenberg, 1841
Cyphoderia margaritacea Schlumberger, 1845
Euglypha curvata Perty, 1852
Lagynis baltica Schultze, 1854
Euglypha ampulla Claparède and Lachmann, 1859
Euglypha baltica Wallich, 1864
Euglypha margaritacea Wallich, 1864
Difflugia Ehrenberg, 1869
Difflugia adunca Ehrenberg, 1871
Difflugia alabamensis Ehrenberg, 1871
Difflugia uncinnata Ehrenberg, 1871
Difflugia margaritacea Ehrenberg, 1871

##### Distribution

**Pirin Mt.** (Golemansky 1974, new data); **Rhodopes Mt.** (Pateff 1924, Golemansky et al. 2006; Todorov et al. 2009, Heger et al. 2010); **Rila Mt.** (Golemansky and Todorov 1993, Todorov and Golemansky 2000, Todorov 2004, Todorov 2005, Todorov et al. 2009, new data); **Stara Planina Mt.** (new data); **Vitosha Mt.** (Pateff 1924, Golemansky 1965, Golemansky and Todorov 1985, Golemansky and Todorov 1990, Todorov 1993, Todorov and Golemansky 1995, Todorov et al. 2009, Heger et al. 2010, new data).

#### Cyphoderia major

(Penard, 1891)

Cyphoderia margaritacea var. major Penard, 1891

##### Distribution

**Pirin Mt.** (new data); **Rila Mt.** (Todorov et al. 2009, Heger et al. 2010, new data); **Stara Planina Mt.** (new data); **Vitosha Mt.** (new data).

#### Campascus

Leidy, 1879

#### Campascus minutus

Penard, 1899

Campascus triqueter var. minuta Awerintzew, 1906

##### Distribution

**Rhodopes Mt.** (Golemansky et al. 2006); **Rila Mt.** (Todorov and Golemansky 2000, new data); **Stara Planina Mt.** (new data); **Vitosha Mt.** (Golemansky 1965, Golemansky and Todorov 1990, new data).

#### Campascus triqueter

Penard, 1891

##### Distribution

**Rila Mt.** (Golemansky and Todorov 1993, Todorov and Golemansky 2000); **Vitosha Mt.** (Golemansky 1965, Golemansky and Todorov 1990,Todorov 1993, Todorov and Golemansky 1995).

#### Paulinellidae

de Saedeller 1934, emend. Adl et al. 2012

#### Paulinella

Lauterborn, 1895

#### Paulinella chromatophora

Lauterborn, 1895

##### Distribution

**Rhodopes Mt.** (Golemansky et al. 2006); **Rila Mt.** (Todorov 2004).

#### INCERTAE SEDIS euglyphid testate amoebae

#### Tracheleuglypa

Deflandre, 1928

#### Tracheleuglypa acolla

Bonnet et Thomas, 1955

##### Distribution

**Pirin Mt.** (new data); **Rhodopes Mt.** (Golemansky et al. 2006); **Rila Mt.** (Todorov and Golemansky 2000, Todorov 2005, new data); **Stara Planina Mt.** (new data); **Vitosha Mt.** (Todorov 1993, Todorov and Golemansky 1995, new data).

#### Tracheleuglypa dentata

(Moniez, 1888) Deflandre, 1928

Sphenoderia lenta Leidy, 1879 (in part)
Euglypha Vejdovsky, 1882
Euglypha dentata Moniez, 1888
Sphenoderia dentata Penard, 1890

##### Distribution

**Pirin Mt.** (Golemansky 1974, new data); **Rhodopes Mt.** (Golemansky 1968, Golemansky et al. 2006); **Rila Mt.** (Golemansky and Todorov 1993, Todorov and Golemansky 2000, Todorov 2005 new data); **Stara Planina Mt.** (new data); **Vitosha Mt.** (Pateff 1924, Golemansky 1965, Golemansky and Todorov 1985, Golemansky and Todorov 1990, Todorov 1993, Todorov and Golemansky 1995, new data).

##### Notes

The species has been recorded both as nominal species and as synonym *S. dentata* (**Vitosha Mt.**).

#### Chlamydophryidae

de Saedeleer, 1934

#### Lecythium

Hertwig and Lesser, 1874

#### Lecythium mutabilis

(Bailey, 1853)

Pamphagus mutabilis Bailey, 1853
Pamphagus avidus Leidy, 1879
Lecythium mutabile Wailes, 1915

##### Distribution

**Rila Mt.** (Pateff 1924).

##### Notes

The species has been recorded as synonym *P. mutabilis* (**Rila Mt.**).

#### Pseudodifflugiidae

de Saedeleer, 1934

#### Pseudodifflugia

Schlumberger, 1845

#### Pseudodifflugia fascicularis

Penard, 1902

##### Distribution

**Rhodopes Mt.** (Golemansky 1968, Golemansky et al. 2006); **Rila Mt.** (Pateff 1924).

#### Pseudodifflugia gracilis

Schlumberger, 1845

Pleurophrys sphaerica Claparède and Lachmann, 1858
Pleurophrys angulata Mereschkovsky, 1879

##### Distribution

**Rhodopes Mt.** (Golemansky et al. 2006); **Rila Mt.** (Golemansky and Todorov 1993, Todorov and Golemansky 2000).

## Discussion

Prior to our investigation, the number of known *Sphagnum*-dwelling testate amoebae in Bulgaria was 155. Our study increases this number with 16 species and the present checklist comprises 171 species classified into 43 genera, 20 families, three orders, three classes and three phyla. We present data for 16 new *Sphagnum*-dwelling testate amoebae in Bulgaria and new distribution data for 134 species. Of them, 99 species are recorded from Stara Planina Mt., for which there was no available data to date. Additionally we report 69 new species for Pirin Mt., 21 for Vitosha Mt. and 18 for Rila Mt. Thirty six species have been synonymised according to the latest taxonomic changes. All known synonyms of valid names are also listed. The species *Euglypha brachiata* Penard, 1902 and the infrasubspecific taxa Difflugia compressa var. africana Gauthier-Lièvre et Thomas, 1958 have erroneously been recorded, because the descriptions of the found individuals fully correspond to *E. acanthophora* and *Z. compressa*, respectively. These misidentified taxa have been transferred to valid species. Three of the recorded species have not been included in the checklist, because they are currently not refering to testate amoebae (*Cochliopodium bilimbosum* and *C. echinatum* are naked amoebae and *Microgromia elegantula* is freshwater foraminifera).

About 30% (51 species) of all recorded testate amoebae are typical sphagnicolous inhabitants, which only exceptionally can be found in other habitats. These are mostly the representatives of the genera *Nebela* (5 species), *Sphenoderia* (5), *Lesquereusia* (4), *Longinebela* (4), *Padaungiella* (4), *Quadrulella* (4), *Heleopera* (3) etc. The most numerous (67 species, 39%) are the species which are found in *Sphagnum* mosses but are also common inhabitants of freshwater basins. The genera *Difflugia* (28), *Centropyxis* (7) and *Arcella* (6) have the largest number of representatives in this group. Most likely, some of these species are not usual inhabitants and fall incidentally into the *Sphagnum* mosses, which develop around the shores of freshwater basins (e.g. *Difflugia lobostoma, D.microclaviformis, D.urceolata, Netzelia tuberculata* etc.). The relative share of species that are rather inhabitants of soils and soil mosses, but also occur in peatlands is comparatively smaller (about 18%, 31 species). Most of them rarely occur and usually have a low population density in *Sphagnum* mosses (e.g. *Awerintzewia cyclostoma, Bullinularia indica, Centropyxis cryptostoma, Centropyxis orbicularis, Cyclopyxisarceloides, Cyclopyxis puteus, Heleoperasylvatica, Padaungiella wailesi, Plagiopyxis callida, Plagiopyxis labiata, Trinema galeata* etc.). The number of eurybiotic species, which are widely distributed in diverse habitats, including peatlands, is the smallest. Twenty one species (about 12% of all recorded) can be referred to eurybionts. Of these, the most frequently occurring in *Spagnum* mosses are *Centropyxis aerophila, Corythion dubium, Cyclopyxis eurystoma, Euglypha laevis, Phryganella acropodia, Trinema lineare* etc.

On a generic level, the genera *Difflugia* (32), *Centropyxis* (17), *Euglypha* (13), *Arcella* (10) and *Nebela* (8) have the greatest species richness. These five genera constitute about 47% of all testate amoebae recorded in *Sphagnum* mosses in Bulgaria. Of the other genera, 23 are represented with 2-5 species and 15 with one species only.

From families, the largest richness have Difflugiidae (39 species, 4 genera), Hyalospheniidae (26, 7), Centropyxidae (17, 1), Euglyphidae (13, 1) and Arcellidae (10, 1), which together make up two thirds of *Sphagnum*-dwelling testate amoebae in Bulgaria. Six families are represented by one species of one genus only (Microcoyciidae, Microchlamyidae, Netzeliidae, Amphitremidae, Paulinellidae and Chlamydophryidae).

At a higher taxonomic level, the most numerous is the order Arcellinida, which includes 128 species of 28 genera and 11 families, followed by the order Euglyphida with 42 species of 14 genera and 8 families and finally by the order Amphitremida with one species of 1 genus and 1 family only.

## Supplementary Material

Supplementary material 1 Checklist of *Sphagum*-dwelling testate amoebae from Bulgaria_Distribution data Data type: Distribution data
Brief description: Data for the distribution of testate amoebae in *Sphagnum* mosses in Bulgaria on the basis of literature and of additional data, obtained in our research over the past two years.
File: oo_193063.xlsx Nikola Bankov, Milcho Todorov and Anna Ganeva

Supplementary material 2 Sampling sites information Data type: Sampling sites data
Brief description: Data concerning sampling sites, including date, region, altitude, coordinates, *Sphagnum* moss species, as well as many physical and chemical parameters of the sampling sites.
File: oo_190421.xlsx Nikola Bankov, Milcho Todorov and Anna Ganeva

## Figures and Tables

**Figure 1a.**
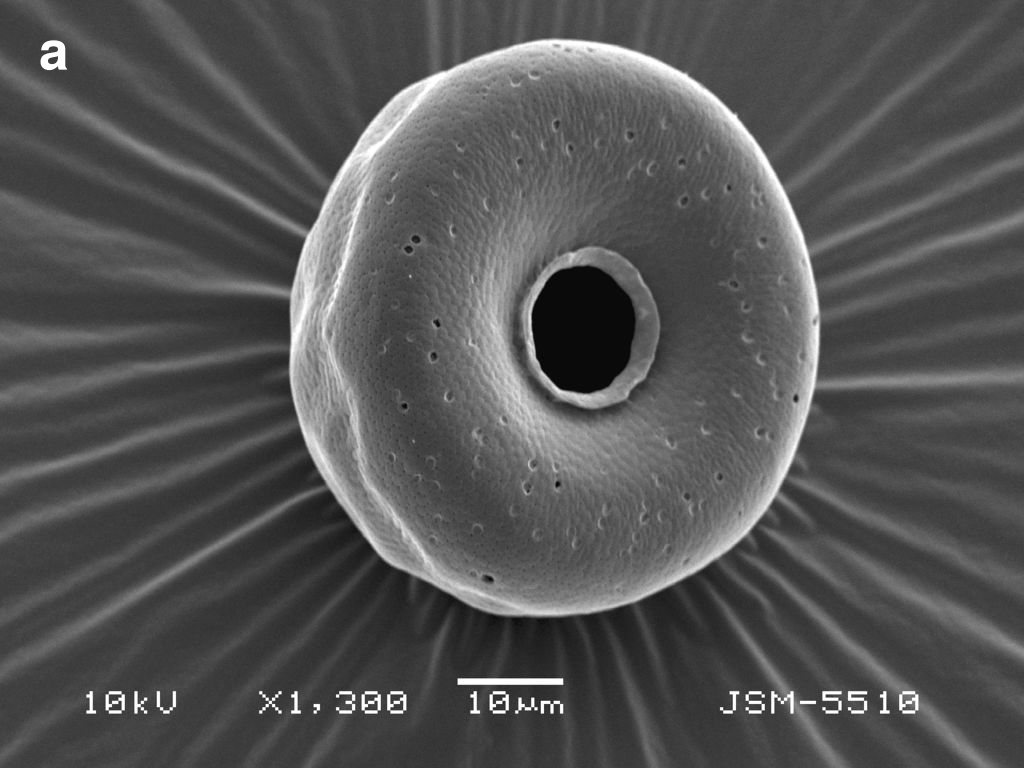
*Arcella intermedia* (Deflandre) (Arcellidae)

**Figure 1b.**
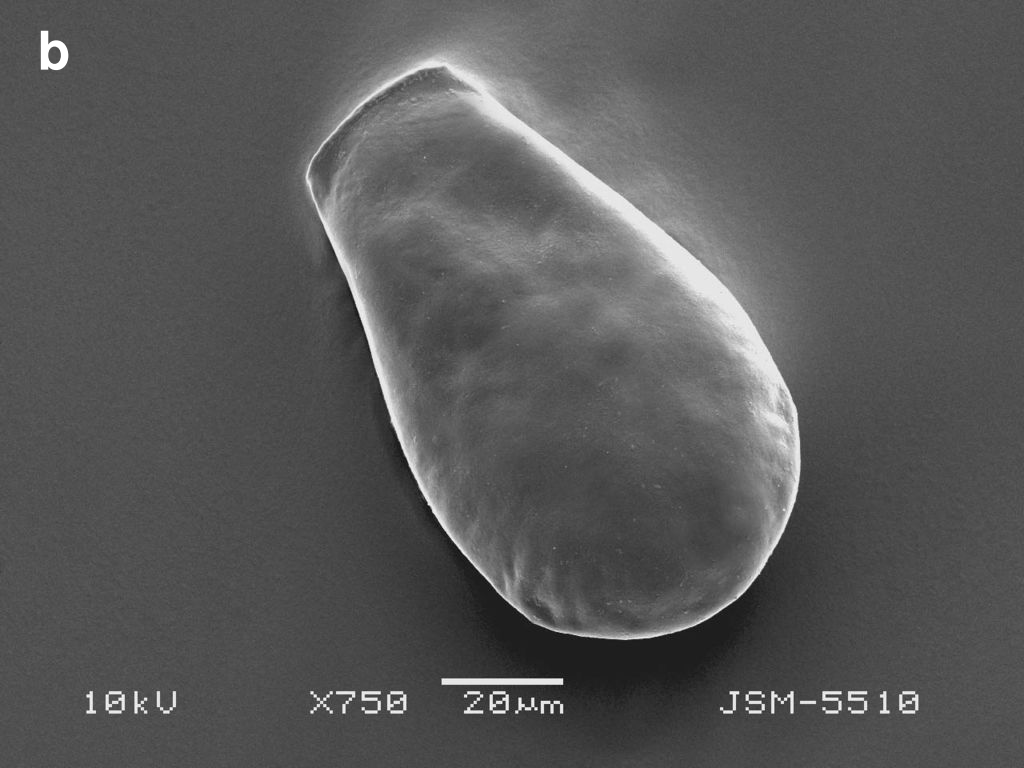
*Hyalosphenia papilio* (Leidy) (Hyalospheniidae)

**Figure 1c.**
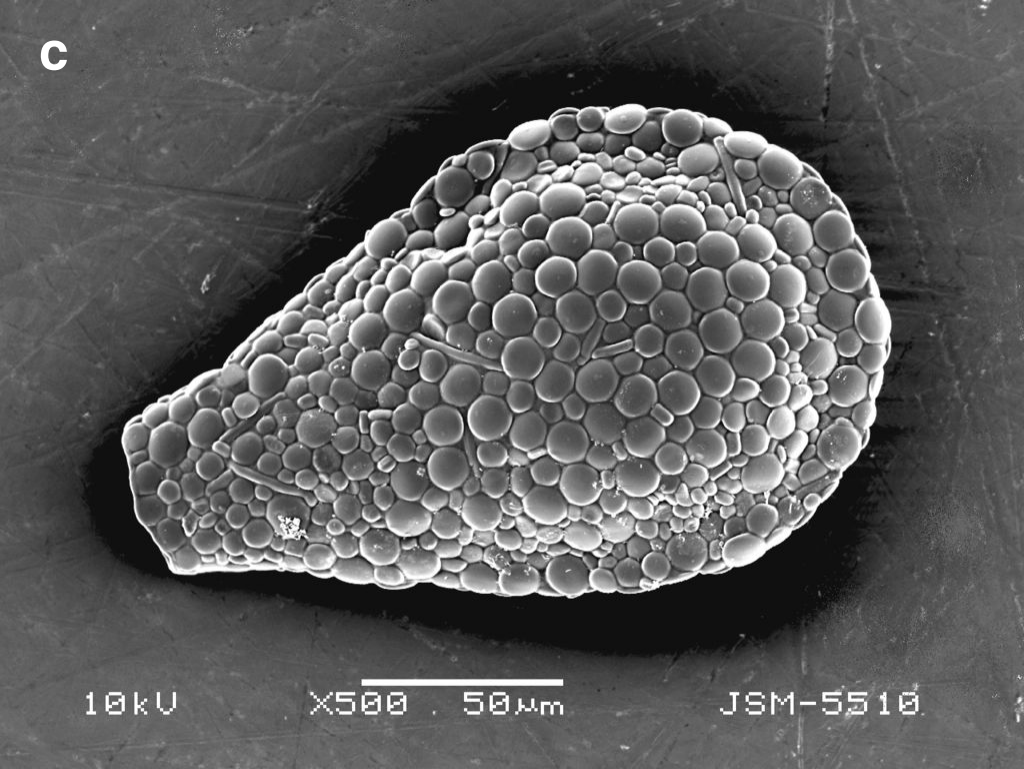
*Gibbocarina galeata* (Penard) (Hyalospheniidae)

**Figure 1d.**
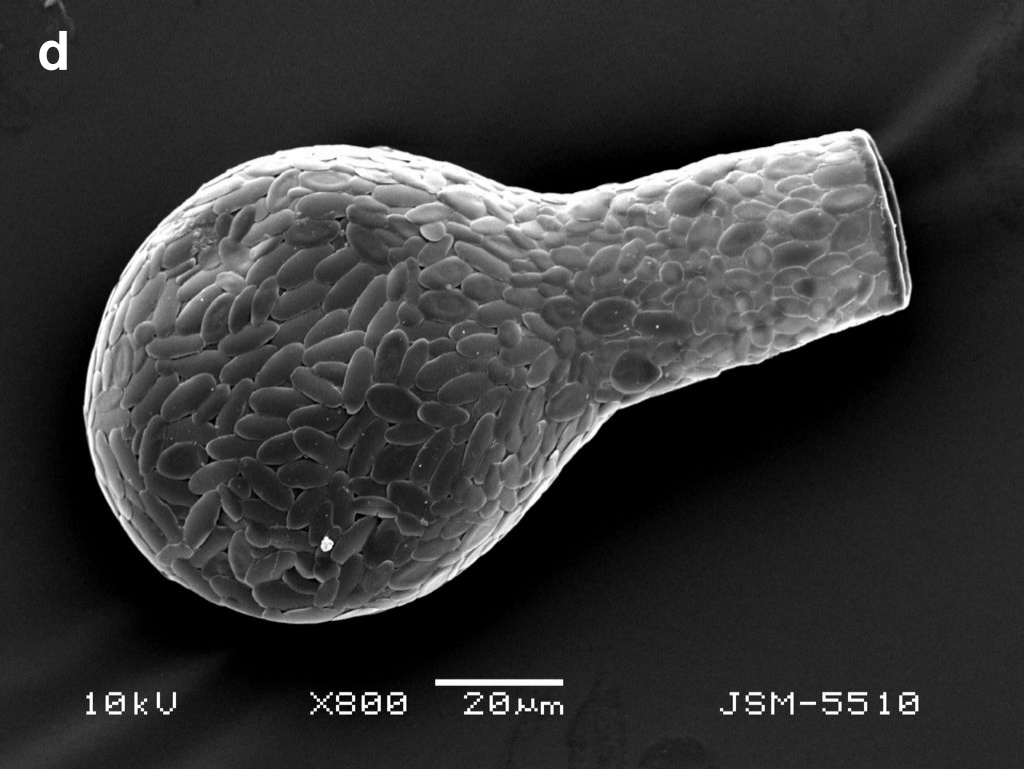
*Padaungiella lageniformis* (Penard) (Hyalospheniidae)

**Figure 1e.**
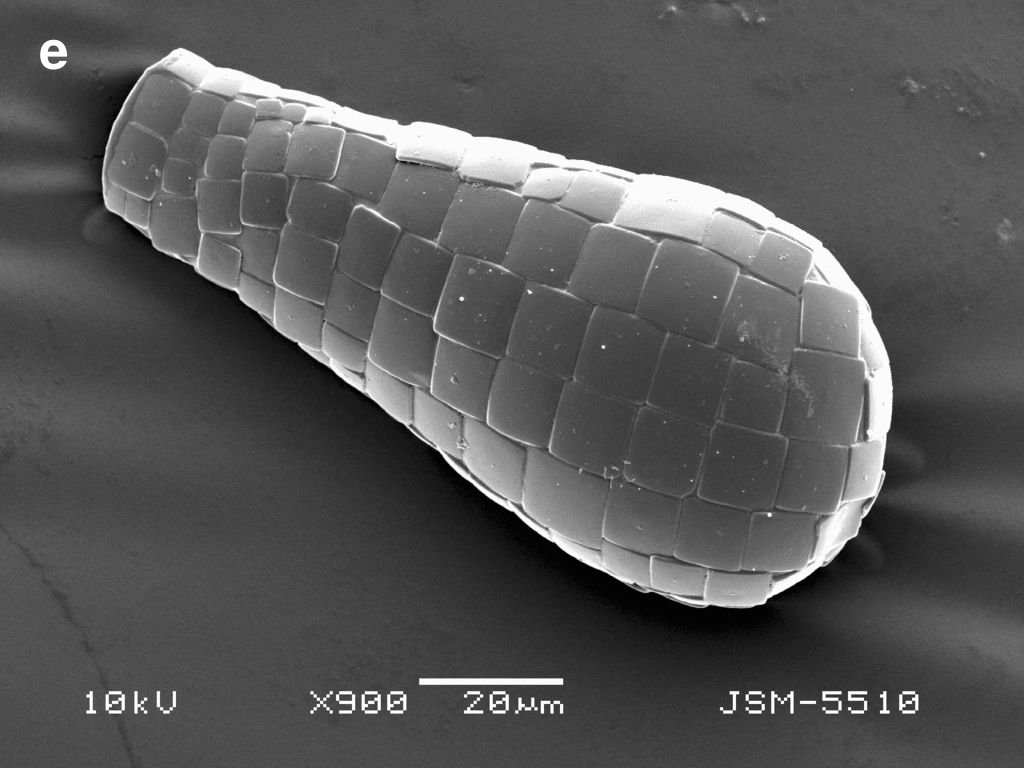
*Quadrulella longicollis* Taranek (Hyalospheniidae)

**Figure 1f.**
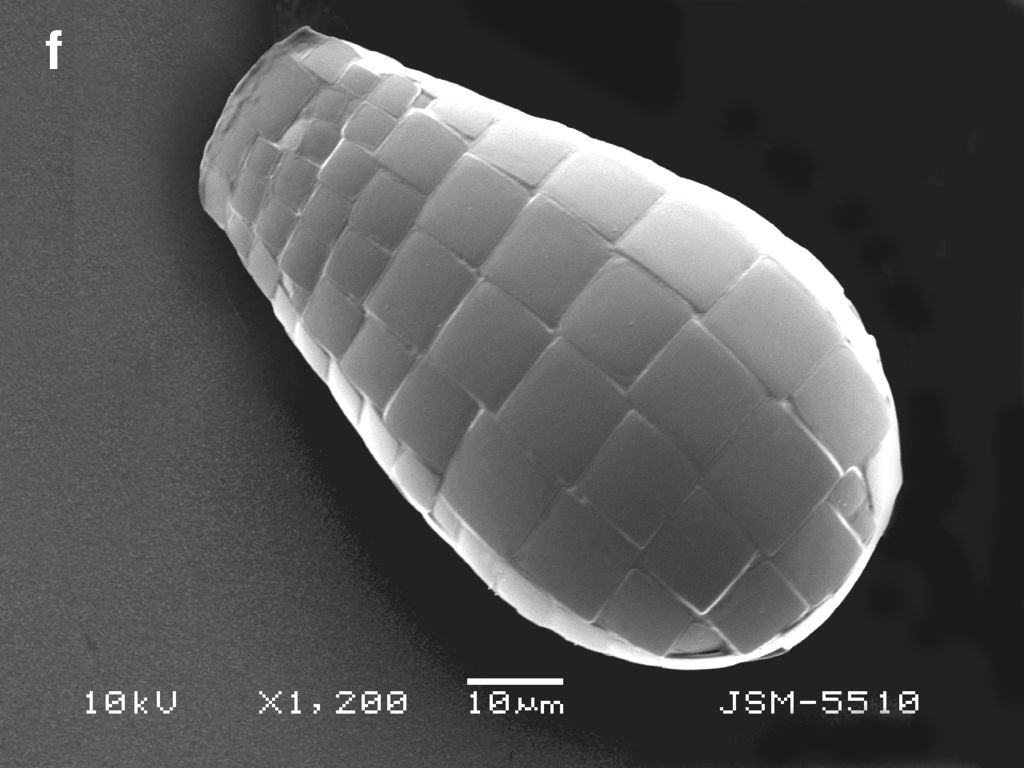
*Quadrulella symmetrica* (Wallich) (Hyalospheniidae)

**Figure 2a.**
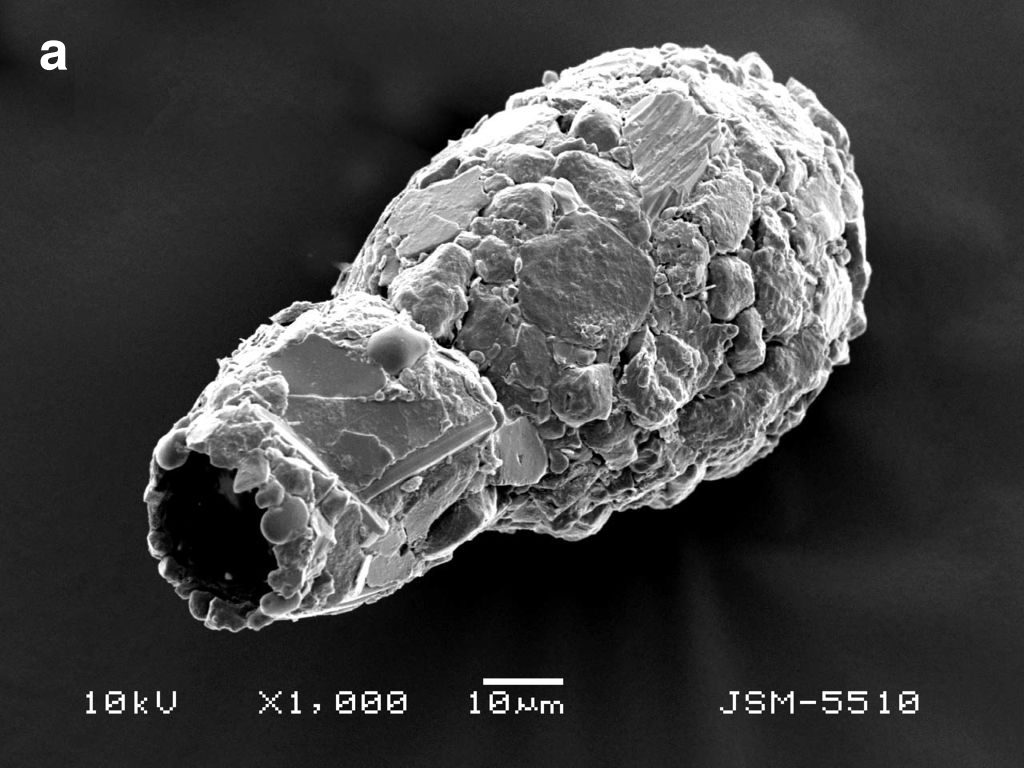
*Lagenodifflugia bryophila* (Penard) (Difflugiidae)

**Figure 2b.**
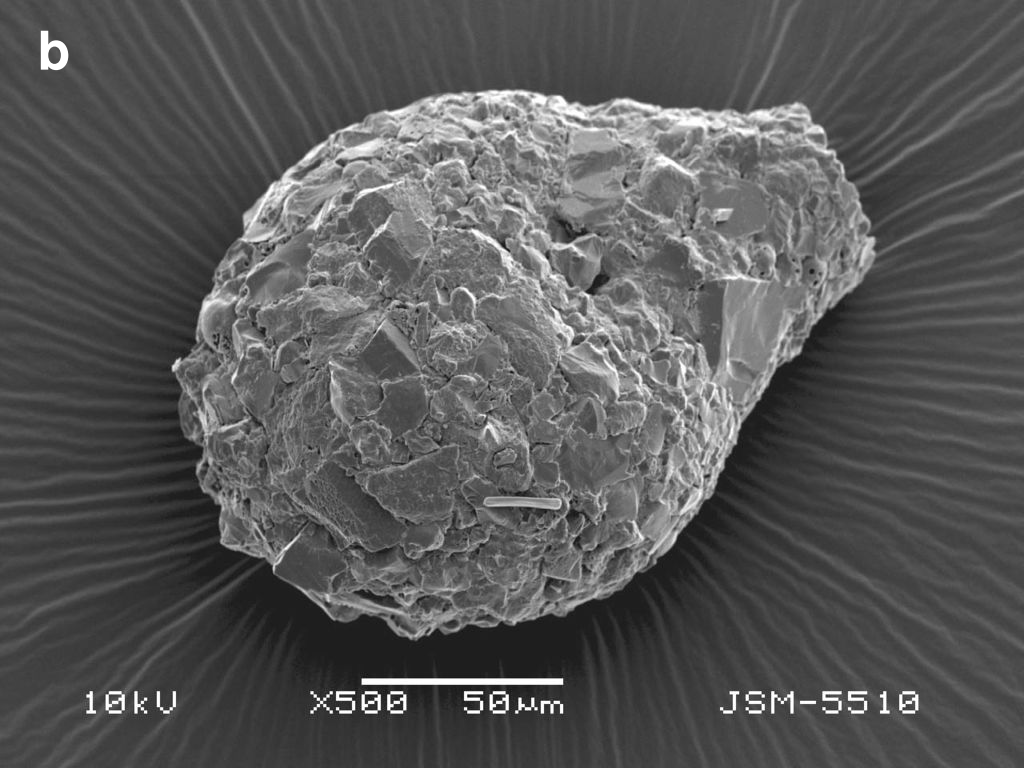
*Zivkovicia compressa* (Carter) (Difflugiidae)

**Figure 2c.**
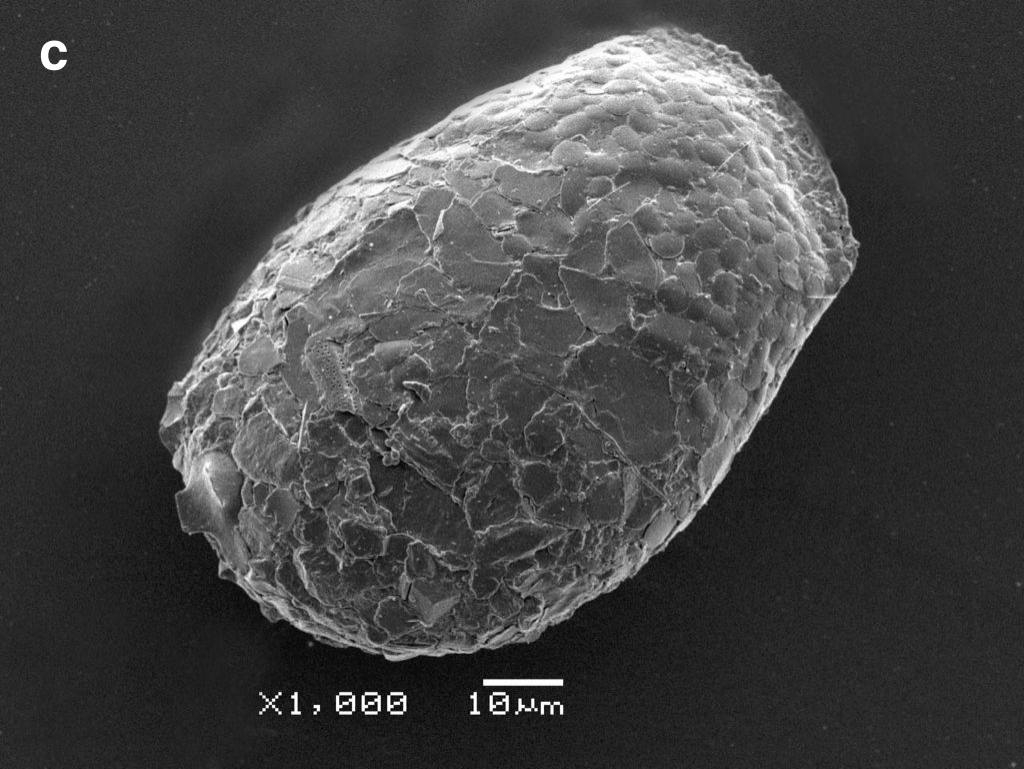
*Heleopera rosea* Penard (Incertae sedis Arcellinida)

**Figure 2d.**
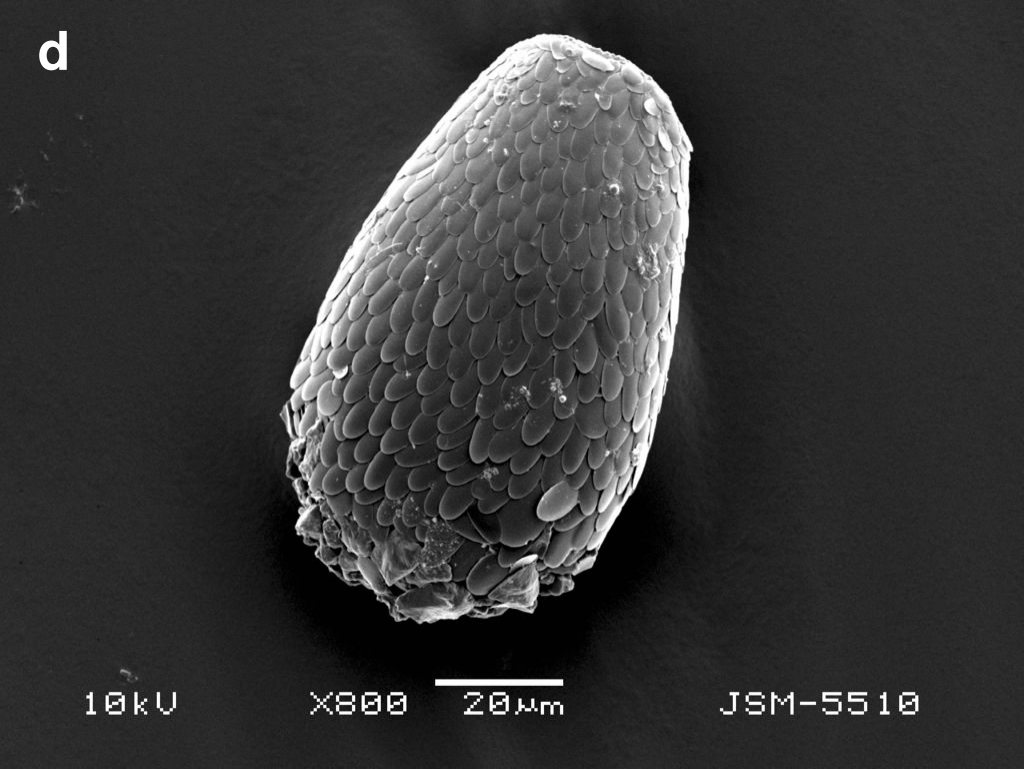
*Heleopera petricola* Leidy (Incertae sedis Arcellinida)

**Figure 2e.**
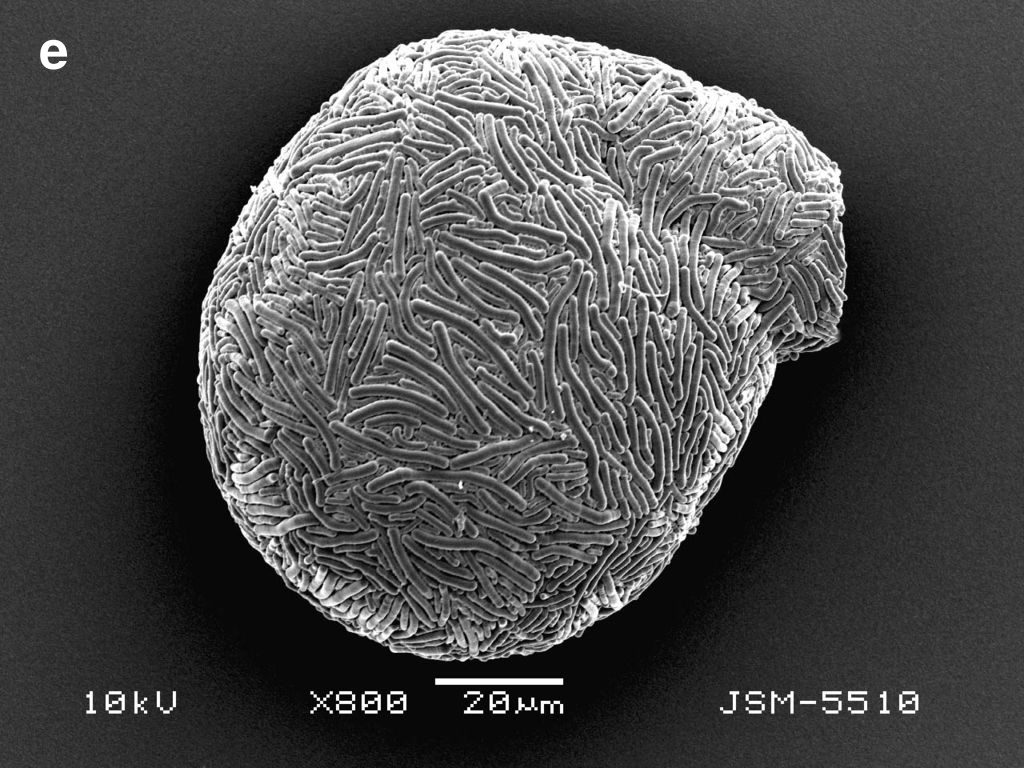
*Lesquereusia spiralis* (Ehrenberg) (Incertae sedis Arcellinida)

**Figure 2f.**
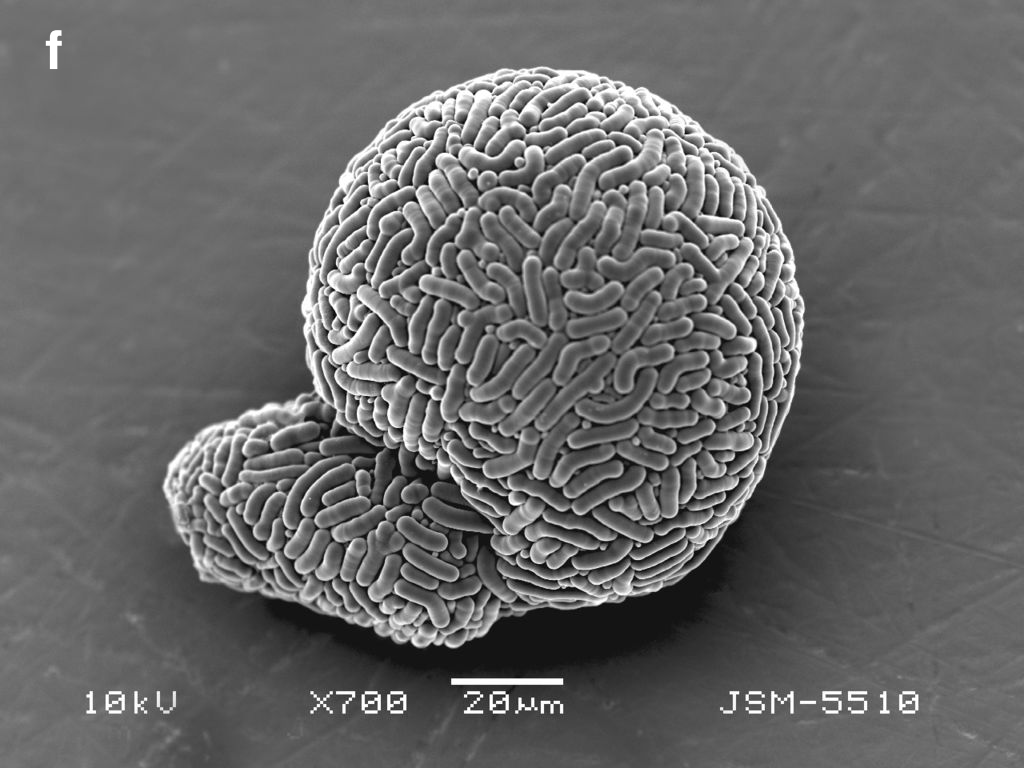
*Lesquereusia epistomium* Penard (Incertae sedis Arcellinida)

**Figure 3a.**
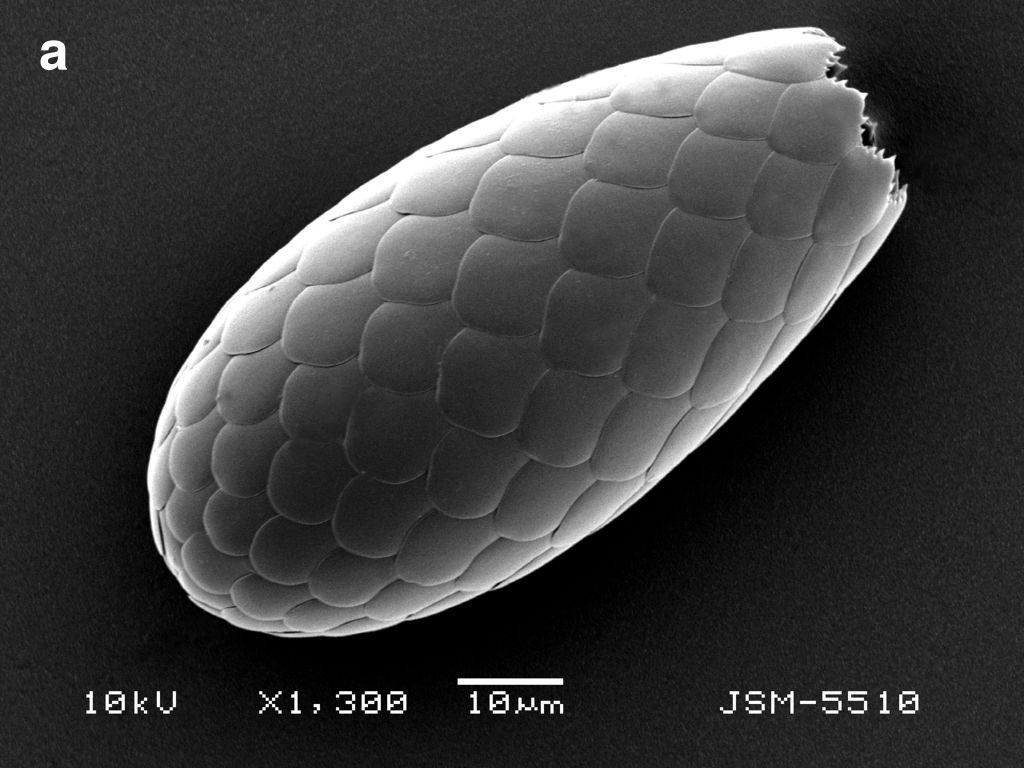
*Euglypha tuberculata* Dujardin (Euglyphidae)

**Figure 3b.**
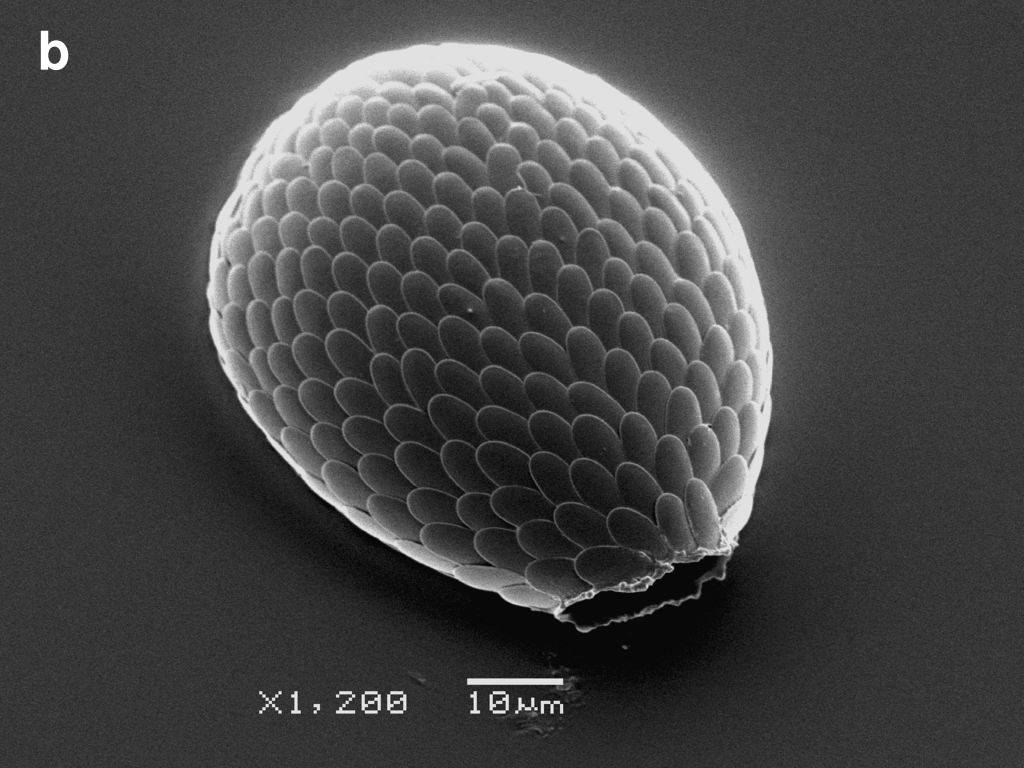
*Assulina seminulum* (Ehrenberg) (Assulinidae)

**Figure 3c.**
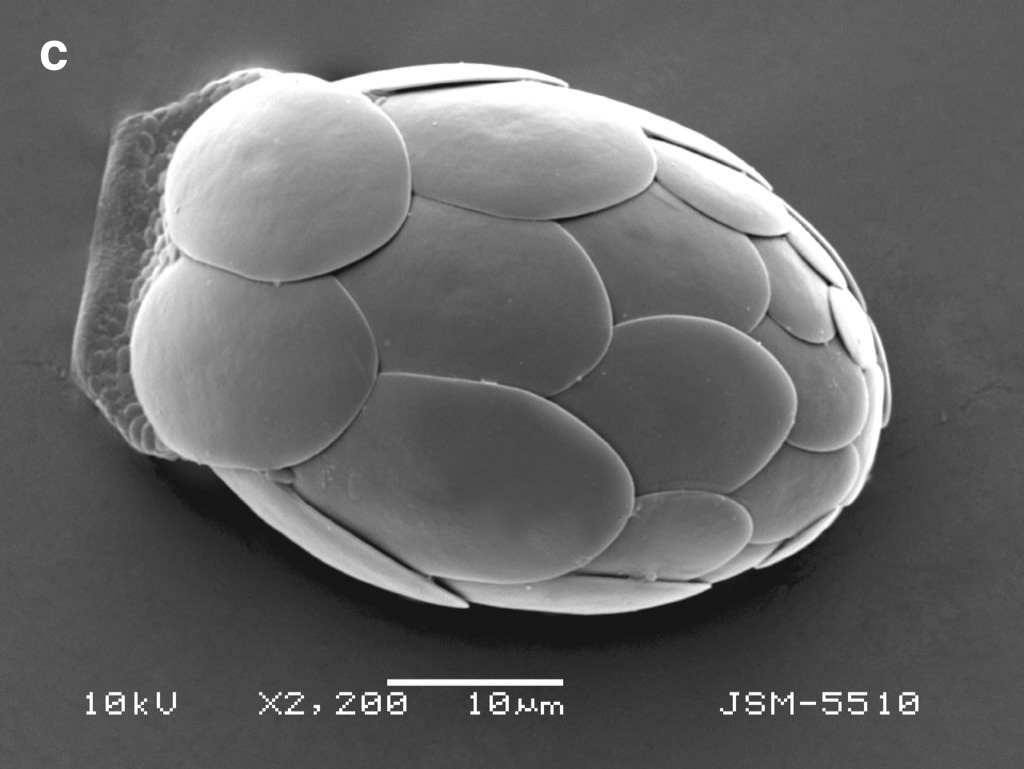
*Sphenoderia fissirostris* Penard (Sphenoderiidae)

**Figure 3d.**
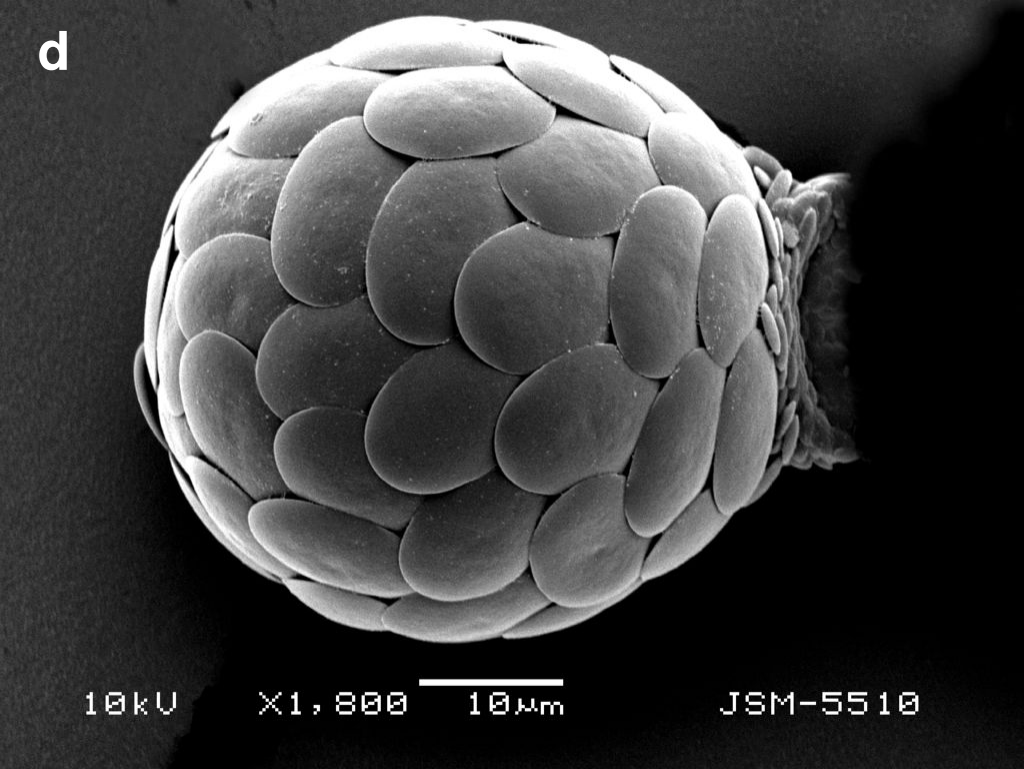
*Sphenoderia lenta* Schlumberger (Sphenoderiidae)

**Figure 3e.**
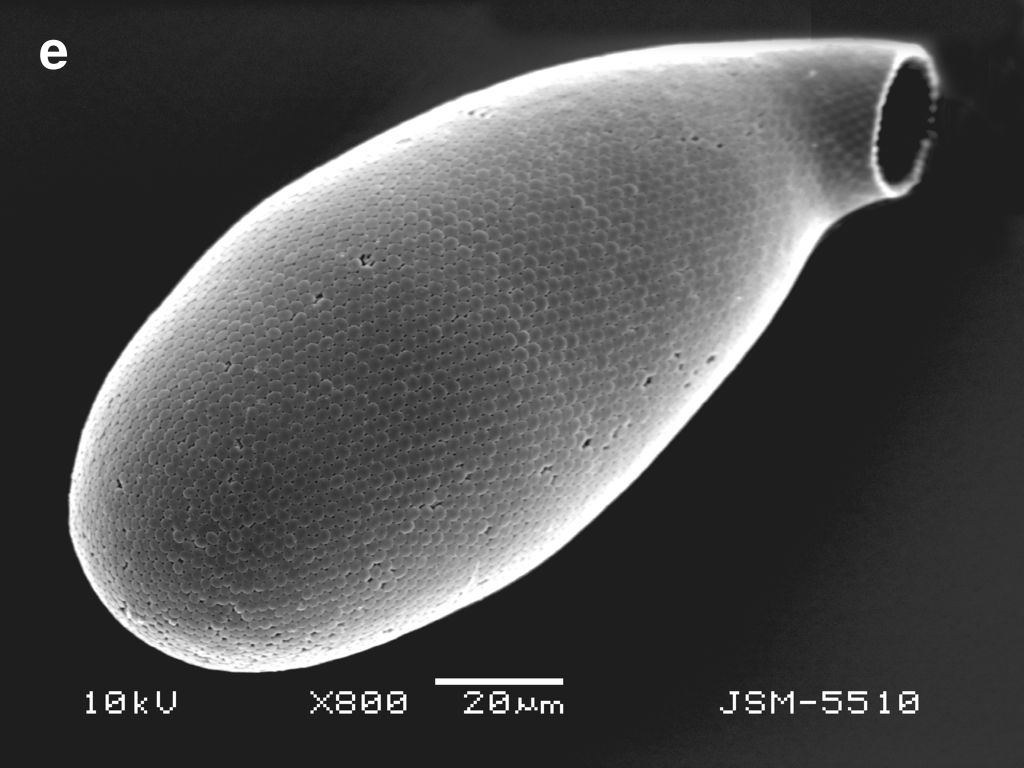
*Cyphoderia major* (Penard) (Cyphoderiidae)

**Figure 3f.**
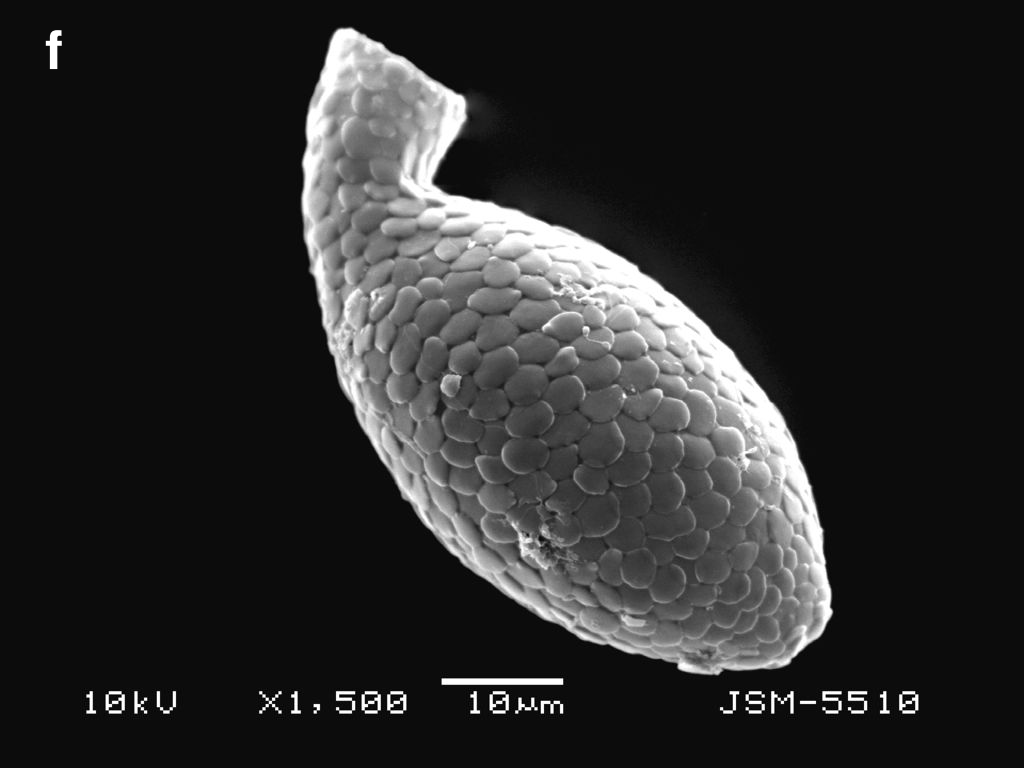
*Campascus minutus* Penard (Cyphoderiidae)

**Figure 4a.**
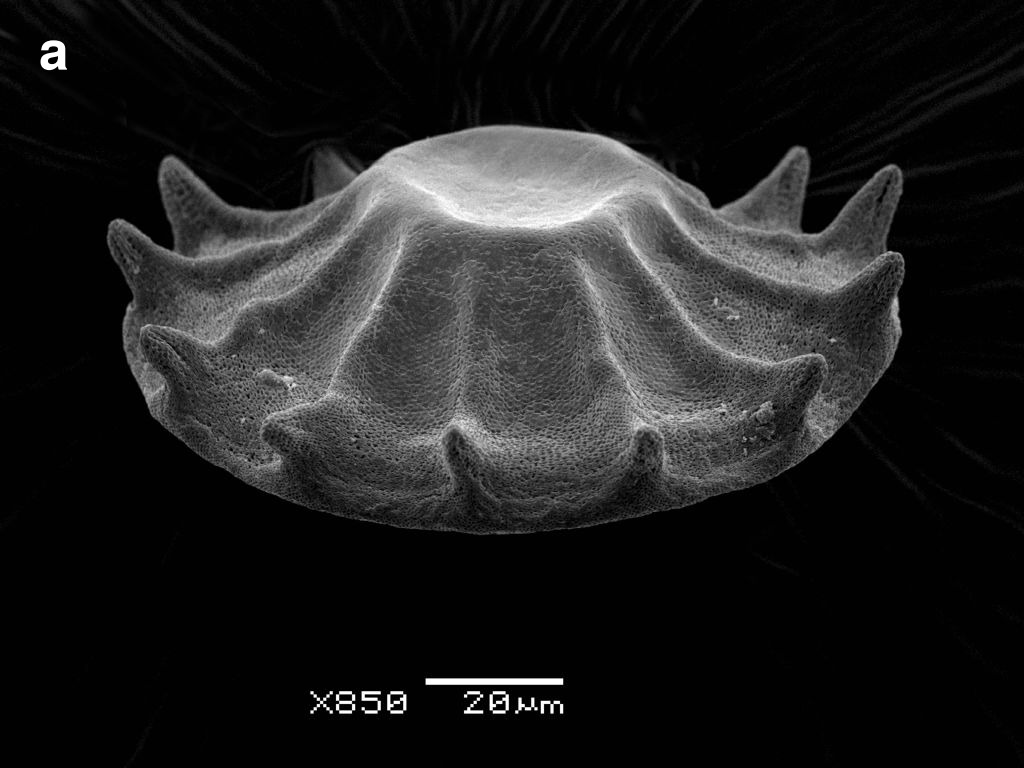
*Arcella dentata* Ehrenberg (Arcellidae)

**Figure 4b.**
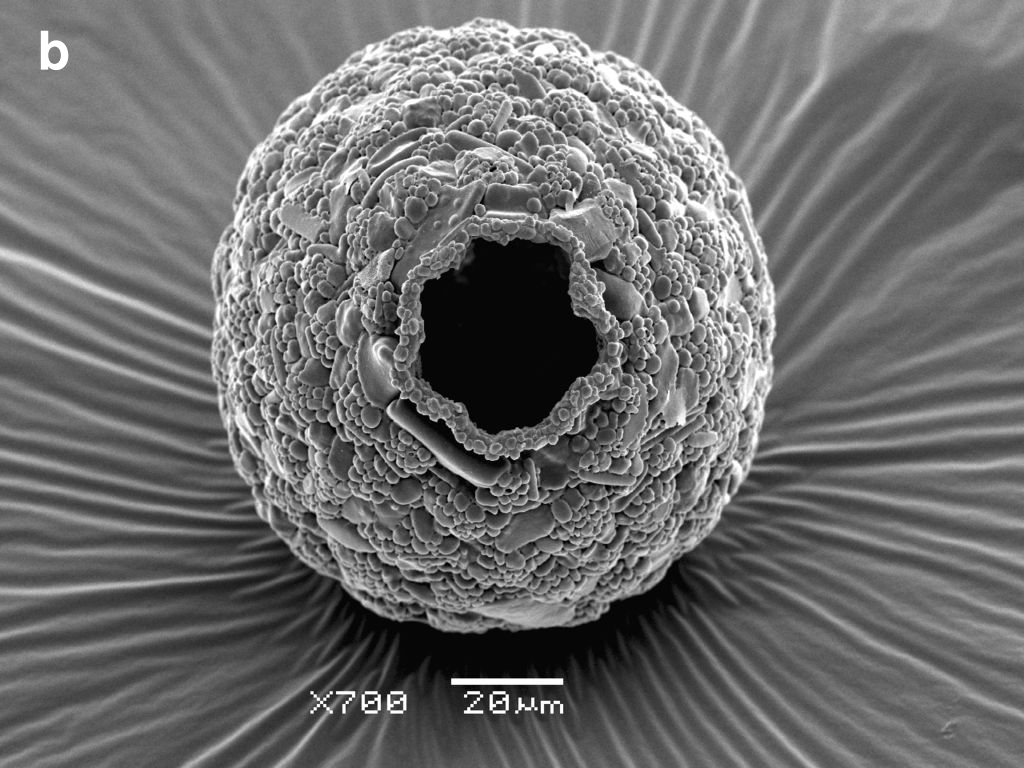
*Netzelia tuberculata* (Wallich) (Netzeliidae)

**Figure 4c.**
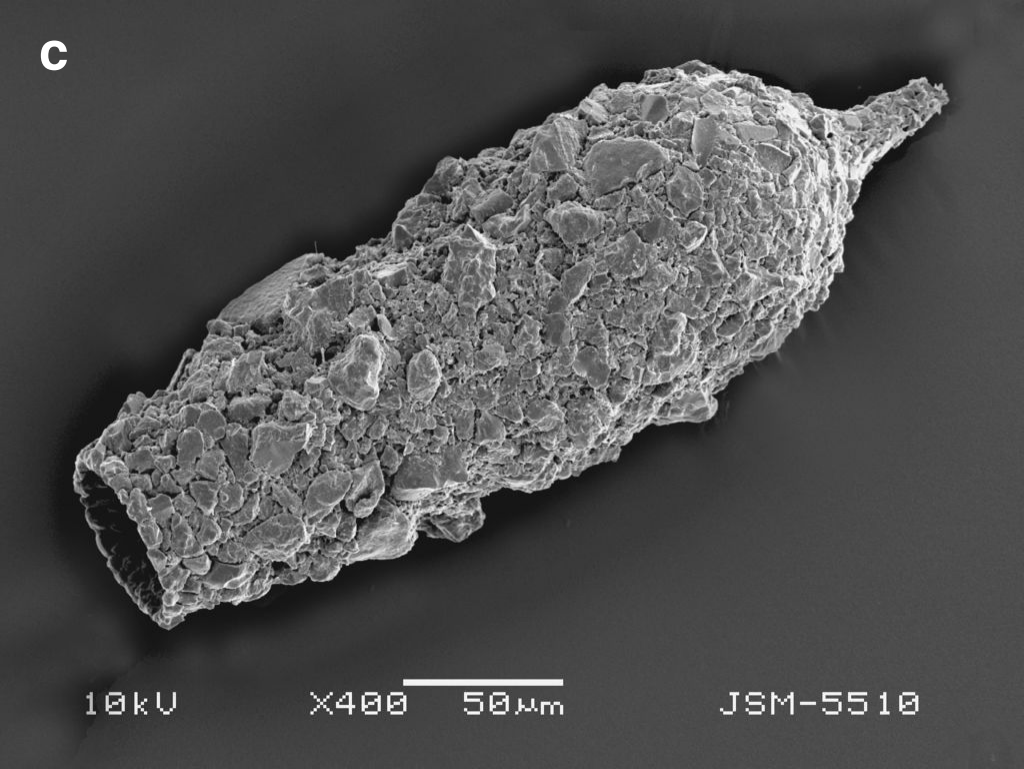
*Difflugia acuminata* Ehrenberg (Difflugiidae)

**Figure 4d.**
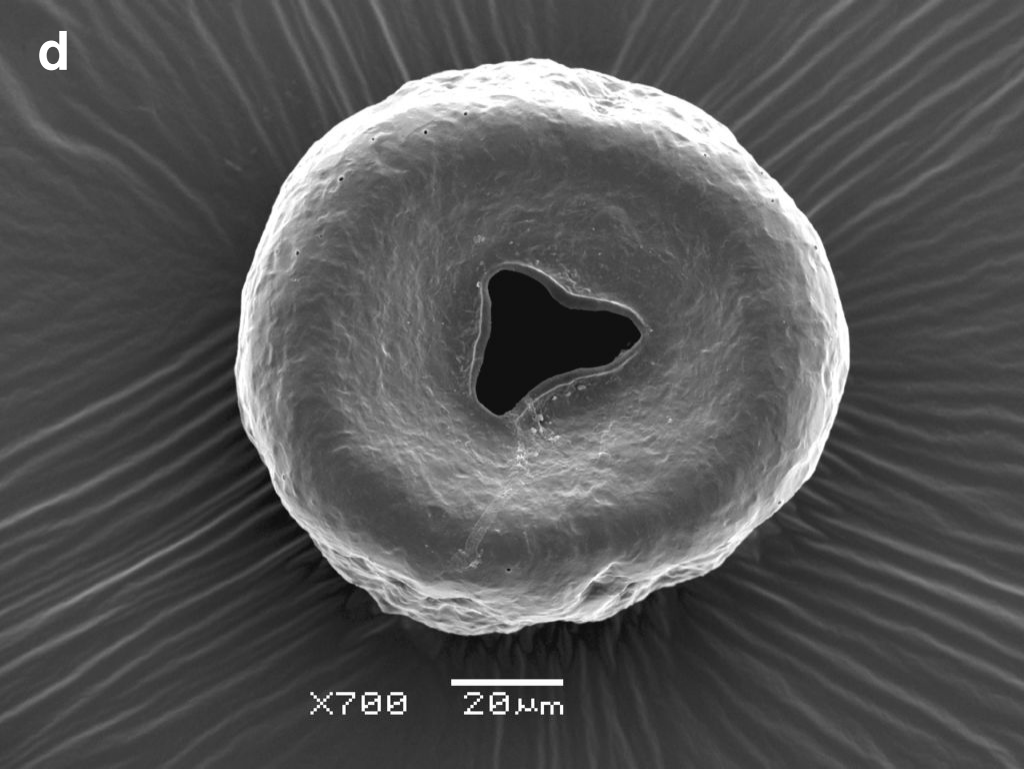
*Trigonopyxis arcula* (Leidy) (Trigonopyxidae)

**Figure 4e.**
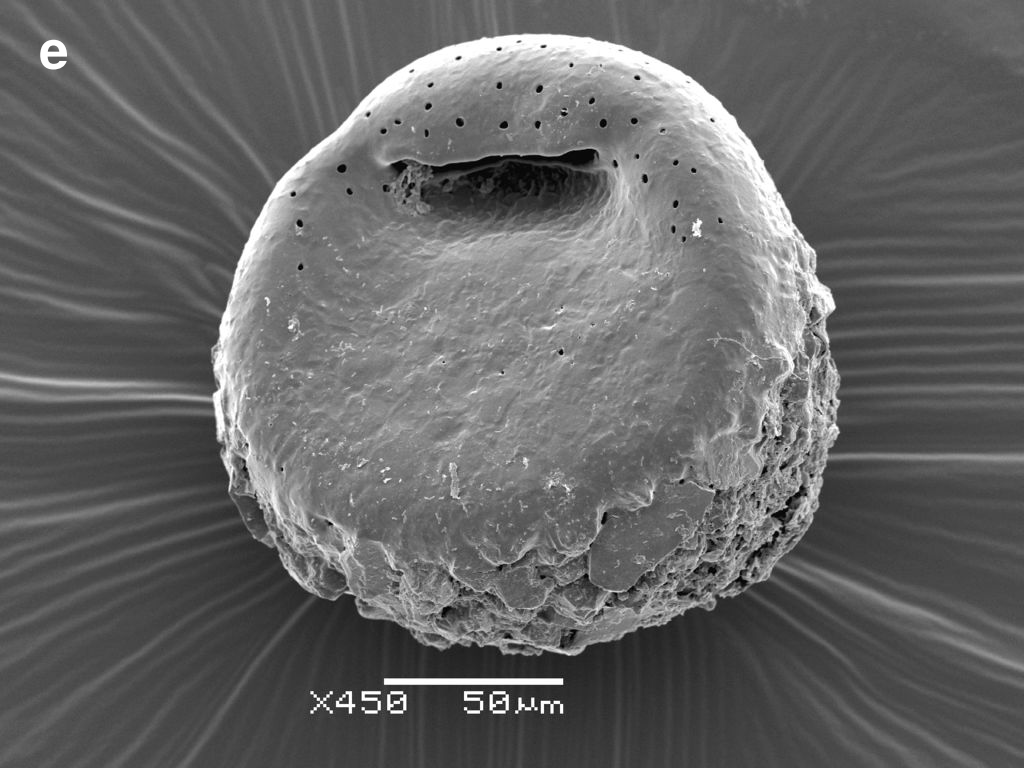
*Bullinularia indica* (Penard) (Plagiopyxidae)

**Figure 4f.**
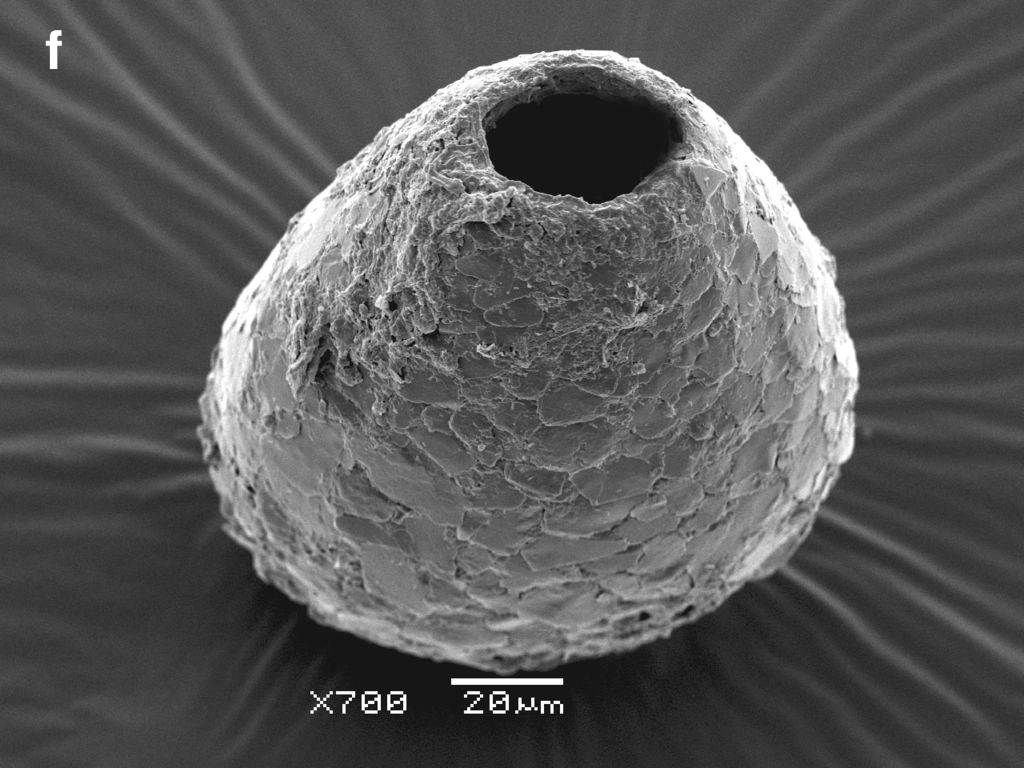
*Awerintzewia cyclostoma* (Penard) (Incertae sedis Arcellinida)
